# Genetic Analysis of Four Sexual Differentiation Process Proteins (isp4/SDPs) in *Chaetomium thermophilum* and *Thermomyces lanuginosus* Reveals Their Distinct Roles in Development

**DOI:** 10.3389/fmicb.2019.02994

**Published:** 2020-01-06

**Authors:** Xiang-Li Xie, Yi Wei, Yan-Yue Song, Guan-Ming Pan, Li-Na Chen, Gang Wang, Shi-Hong Zhang

**Affiliations:** ^1^College of Plant Sciences, Jilin University, Changchun, China; ^2^School of Life Sciences, Henan University, Kaifeng, China

**Keywords:** isp4/SDP (sexual differentiation process protein), OPT (oligopeptide transporter), sexual reproduction, 72nt-insertion fragment (72INS), *C. thermophilum*, *T. lanuginosus*

## Abstract

Fungal sexual development requires the involvement of a large number of functional genes. Fungal genes encoding sexual differentiation process proteins (*SDPs*), *isps*, have been known for decades. *isp4*/*SDP* and its homologs function as oligopeptide transporters (OPTs), yet their roles in reproduction are unknown. Here, we genetically analyzed all four *isp4/SDP* homologs in the sexual species *Chaetomium thermophilum* and asexual species *Thermomyces lanuginosus.* Using single gene deletion mutants, we found that *T. lanuginosus SDP* (*TlSDP*) participated in asexual sporulation, whereas the other homologs participated in sexual morphogenesis. In complementary tests, *C. thermophilum SDPs (CtSDP1*-*3)* restored sporulation defects in *TlSDP* deletion strains (Δ*TlSDP*), and their translated proteins, which were localized onto the cytomembrane, possessed OPT activity. Interestingly, *CtSDP2* accumulated at the top of the hyphae played a distinct role in determining the sexual cycle, glutathione transport, and lifespan shortening. A unique 72nt-insertion fragment (72INS) was discovered in *CtSDP2*. Biological analysis of the 72INS deletion and DsRED-tagged fusion strains implied the involvement of 72INS in fungal growth and development. In contrast to *TlSDP*, which only contributes to conidial production, the three *CtSDP*s play important roles in sexual and asexual reproduction, and *CtSDP2* harbors a unique functional 72INS that initiates sexual morphogenesis.

## Introduction

Ascomycota, the largest fungal phylum, is mostly defined according to sexual morphological features, particularly the sexual structures in which ascospores are formed (Cavalier-Smith, 2010). Sexual and asexual reproduction are common in ascomycota to produce offspring, but they have completely different features. During asexual reproduction, conidia are generally created from the tips of conidiophores, which are morphologically similar to vegetative hyphae. On the contrary, sexual reproduction of fungi is quite complex. Aggregation of vegetative mycelia indicates the initiation of sexual reproduction. Related hyphal cells differentiate into sterile and fertile hyphal cells, and finally the fruiting mycelium mature hyphae into the ascocarp. The ascogonia and antheridia are two key structures during sexual sporogenesis Hook-shaped structures known as croziers determine the formation of ascospores in the dikaryotic stage of filamentous fungi (Sautter and Hock, 1982). Interestingly, sexual reproduction can be triggered in supposedly asexual fungi, such as *Candida albicans* and *Trichoderma reesei* (Taylor et al., 1999; Magee and Magee, 2004; Seidl et al., 2009; Dyer and O’Gorman, 2011; Dyer and O’Gorman, 2012). *Penicillium chrysogenum*, which has been considered asexual for more than 100 years, has been shown to reproduce sexually based on *MAT* gene organization (Böhm et al., 2013).

Some genes associated with sexual differentiation, including *lsdA*, *nsdD*, and *veA*, have been successfully characterized (Lee et al., 2001; Kim et al., 2002; Han et al., 2010; Palmer et al., 2013). Sexual differentiation process proteins gene (*SDPs*) have been studied extensively in the fission yeast *Schizosaccharomyces pombe* (Sato et al., 1994). In *S. pombe*, five *isp* genes (*isp*3-*isp*7) were identified and they were preferentially expressed during sporogensis. Further study revealed that they were involved in diverse biological/biochemical functions (Yanagida, 2009; Shimanuki et al., 2010; Laor et al., 2014). *Isp4* has been found up-regulated by nitrogen starvation-induced meiosis and found to encode an oligopeptide transporter (OPT). Subsequently, *isp4* homologs have been extensively characterized in yeast (Lubkowitz et al., 1998; Bourbouloux et al., 2000; Hauser et al., 2000; Gonzalez-Lopez et al., 2002; Lubkowitz et al., 2010; Oliver and Joachim, 2010), but only a few OPTs have been reported in filamentous fungi. In *Schizophyllum commune*, the *mtd1* gene is regulated during the mating reaction, and the results of complementation of gene disruption are in accordance with an OPT (Lengeler and Kothe, 1999). In *Colletotrichum gloeosporioides*, *CgOPT1* disruption resulted in fewer spores, reduced pigmentation, and less severe pathogenic effects (Chague et al., 2009). In *Aspergillus fumigatus*, the octuple *OPT* gene deletion mutant that expressed no OPT genes displayed normal growth on various substrates (Thomas et al., 2011). Recently, members of the OPT gene family in *Phanerochaete chrysosporium* and *Ganoderma lucidum* have been analyzed in yeast systems (Xiang et al., 2013, 2017). Although the biochemical transport functions of the OPT genes described above have been confirmed, little is known about their biological roles, particularly in sexual differentiation.

The thermophilic fungi *Thermomyces lanuginosus* and *Chaetomium thermophilum* (McGrath and Varshavsky, 1989) coexist in the same environments, which have relatively high temperatures and abundant lignocellulosic compost. However, the two thermophilic fungi seem to have different reproduction patterns. *T. lanuginosus* reproduces asexually, while *C. thermophilum* reproduces sexually. By analyzing genes up-regulated during sporogenesis, three homologs of yeast *isp4* (Sato et al., 1994) were identified in *C. thermophilum* (termed *CtSDP1–CtSDP3*), and single was identified in *T. lanuginosus* (termed *TlSDP*). We hypothesized that the identified genes were involved in regulating sexual or asexual reproduction in filamentous fungi. To verify this hypothesis and obtain direct insight into the biological roles of the four identified *SDP* homologs, several single gene deletion strains and homologous gene complementation strains were created, and phenotypes related to stages of growth, development, and sporogenesis were analyzed. All *SDP* genes, as expected, were found to be important for sporogenesis of their strains, but only *CtSDP2* was required for sexual morphogenesis. *CtSDP2* appears to determine the formation of sexual structures. We also found a 72nt-insertion fragment in *CtSDP2* and investigated its role in sexual morphogenesis.

## Materials and Methods

### Fungal Strains

*Thermomyces lanuginosus* strain 9W and *Chaetomium thermophilum* strain S4 were isolated from a sample of cow dung compost (CDC) in Jilin province, which is located in northeastern China. Wild-type and mutant strains were cultured at 50°C on potato dextrose agar media (PDA; 200 g/L peeled potato, 20 g/L glucose, and 16 g/L agar) or complete minimal media (CM; 1 g/L yeast extract, 0.5 g/L casein enzymatic hydrolyzate, 0.5 g/L casamino acids hydrolyzate, 10 g/L glucose, 1 g/L Ca(NO_3_)2⋅4H_2_O, 0.2 g/L KH_2_PO_4_, 0.25 g/L MgSO_4_⋅7H_2_O, 0.15 g/L NaCl, and 16 g/L agar).

### Vectors and Construction Strategies

Serial plasmids were employed for genetic transformation. Based on the PXEH vector, a vector containing the upstream and downstream flanking regions of the target gene was reconstructed to accomplish gene-specific deletions. This vector also conferred kanamycin resistance in *Agrobacterium tumefaciens* and *Escherichia coli* and harbored the hygromycin B phosphotransferase (*hyg*) gene as a selection marker for fungal deletion mutants. Briefly, the flanking sequences of the *TlSDP* gene were PCR-amplified from *T. lanuginosus* 9W DNA genome. The left (988 bp) and right (1095 bp) border flanking sequences were amplified and validated. PCR primers were designed to introduce the required enzyme cutting sites; the left flanking sequence was *Xho*I and *Sac*I, and the right flanking sequence was *Bam*HI and *Hin*dIII. Both flanking sequences were digested, purified and ligated into the PXEH vector. The *TlSDP*-PXEH vector was confirmed by PCR, enzyme digestion and sequencing validation, after which it was used for further research. To create single gene deletion mutants of *C. thermophilum* for *CtSDP1*, *CtSDP2*, and *CtSDP3*, the corresponding flanking sequences were amplified from *C. thermophilum* S4 DNA genome. Deletion vectors *CtSDP1*-PXEH, *CtSDP2*-PXEH, and *CtSDP3*-PXEH were reconstructed according to the approach described above. Δ*CtSDP1*, Δ*CtSDP2*, and Δ*CtSDP3* were created by gene replacement (Supplementary Figure S1 and Supplementary Tables S1, S2).

To ensure that the obtained mutant phenotype could be attributed to the desired deletion and to investigate sub-localization of the protein, several single-gene and polygenic complementary vectors were constructed based on vectors such as pKD7-RED, pKAICR80, and pKAFCR100. pKD7-RED contains the *DsRED* gene as a subcellular localization tag and the G418-resistance gene as a selection marker. Plasmids pKAICR80 and pKAFCR100 contain the *DsGFP* gene as a tag and neomycin phosphotransferase II as a selection marker. For the construction of single-gene complementary vectors, the RFP in the vector was removed. The main distinction between the complementary vector and the corresponding sub-localization vector (which contains both the complemented gene and the fluorescent protein tag genes) is the presence or absence of the DsRED tag gene. The same construction strategies and primers were used for both types of vectors.

The pKD7-RED vector was used for single-gene and sub-localization tag gene expression. The *TlSDP* coding sequence was amplified from 9W cDNA sequences, whereas the coding sequences of *CtSDP1*, *CtSDP2*, and *CtSDP3* were amplified from S4 cDNA sequences. The PCR-amplified fragments were digested with restriction enzymes and ligated into vectors digested with the same enzymes. For pKD7-RED-*TlSDP*, pKD7-RED-*CtSDP1* and pKD7-RED-*CtSDP2*, *Xba*I and *Sal*I were used, whereas *Bam*HI and *Sma*I were used for pKD7-RED-*CtSDP3*. Each of the successfully constructed vectors was transformed into the Δ*TlSDP* strain to generate four complemented strains (Δ*TlSDP*/*TlSDP*, Δ*TlSDP*/*CtSDP1*, Δ*TlSDP*/*CtSDP2*, and Δ*TlSDP*/*CtSDP3*). The 72nt-insertion fragment (72INS) was deleted from pKD7-RED-CtSDP2 using La-TAQ PCR amplification, resulting in the reconstructed vector Δ*TlSDP*:[*CtSDP2*Δ72INS-RED] (Supplementary Table S2).

The pKAICR80 vector and the revamped pKAFCR100 vector were used for polygenic expression. The coding sequences of *CtSDP1*, *CtSDP2*, and *CtSDP3* were amplified as described above. Each of the digested fragments was ligated into the same double-digested pKAFCR80 vector, resulting in three transition plasmids: pKAFCR80-*CtSDP1*, pKAFCR80-*CtSDP2*, and pKAFCR80-*CtSDP3*. For pKAFCR80-CtSDP1, *Xbal*I, and *Kpn*I enzyme cutting sites were added to both sides of the *CtSDP1* sequence; for pKAFCR80-*CtSDP2* and pKAFCR80-*CtSDP3*, *Sma*I/*Sac*I and *Xba*I/*Xho*I were added. The transition plasmids were double digested with *Cla*im and *Pmac*I, respectively. The recovered 35S-P-*CtSDP1*-NOS-T fragment was ligated to the revamped pKAFCR100 plasmid and digested with the same enzymes, after which we obtained the pKAFCR100-*CtSDP1* vector. Next, the 35S-P-*CtSDP2*-NOS-T fragment was ligated into the pKAFCR100-*CtSDP1* vector and double-digested with *Cla*im and *Hap*I to obtain the pKAFCR100-*CtSDP1*-*CtSDP*2 vector. Double-gene expression vectors pKAFCR100-*CtSDP1-CtSDP3* and pKAFCR100-*CtSDP2*-*CtSDP3* were constructed in the same manner. Finally, the 35S-P-*CtSDP3*-NOS-T fragment was ligated into the pKAFCR100-*CtSDP1*-*CtSDP2* vector and double-digested with *Cla*I and *Hap*I, resulting in the three-gene expression vector pKAFCR100-*CtSDP1-CtSDP2-CtSDP3.* Using the same strategy, double and triple gene expression vectors were constructed, after which the Δ*TlSDP*/*CtSDP12*, Δ*TlSDP*/*CtSDP13*, Δ*TlSDP*/*CtSDP23*, and Δ*TlSDP*/*CtSDP123* mutant strains were obtained.

The primer pairs used for a gene cloning and vector construction are listed in Supplementary Table S1. All primers were designed using DNAMAN. PCR cloning and analyses were performed using high fidelity fusion polymerase (Fermentas). Restriction enzymes, T4 DNA ligase, SYBR Premix Ex Taq and other DNA-modifying enzymes were used as recommended by the suppliers (TaKaRa, Dalian).

### ATMT Transformation

*Agrobacterium tumefaciens* strain AGL-1 was used for transformation of *T. lanuginosus* strain 9W and *C. thermophilum* strain S4. The transformation process was modified based on a prior study (Khan et al., 2014). AGL-1 carrying a deletion or expression vector was cultured overnight in a rotatory shaker (180 rpm) at 28°C in 10 mL Luria-Bertani (LB) medium with 50 μg/mL rifampicin and 50 μg/mL kanamycin. Subsequently, 2.0 mL of the bacteria solution was centrifuged at 2,400 × *g* for 10 min, after which the precipitate was resuspended in inducible medium supplemented with 200 μM acetosyringone (AS) to achieve an optical density at 600 nm (OD600) of between 0.2 and 0.4, as assessed using a microplate reader (Molecular Devices, Sunnyvale, CA, United States). The medium was cultured with agitation at 180 rpm for 8–10 h at 28°C until an OD600 value of 0.8 was reached. *T. lanuginosus* 9W was cultured on PDA at 50°C for 4–5 days. The conidia were washed from the clones using induction liquid medium (IM) and adjusted to a final concentration of 1 × 10^5^ conidia/mL. *C. thermophilum* S4 was cultured on CM at 50°C for nearly 7 days. The cleistocarp was harvested by scraping the agar surface, allowing the release of ascospores by shock or compression. The ascospores were filtered through four layers of sterile gauze, washed in sterilized distilled water, and adjusted to a final concentration of 1 × 10^5^ spores/mL.

Sterile Hybond N membranes (Amersham Biosciences, Piscataway, NJ, United States) were placed on solid IM + AS plates. The corresponding concentration of *T. lanuginosus* conidia was mixed with the AGL-1 culture in equal volumes, after which 100 μL of the resulting mixture was pipetted onto the plate, spread evenly with a sterilized SS-Spreader, and air-dried. The plates were co-cultured in the dark at 25°C for 2 days. The filters were transferred onto selective PDA medium containing 80 μg/mL hygromycin B at 50°C in the dark until colonies appeared. The expression transformants were selected with 50 μg/mL G418 rather than hygromycin B. The control group was treated with distilled water.

### Bioinformatic Analysis

Nucleotide and protein sequences were downloaded from the Fungal Genomes Database^1^ and analyzed using PubMed online tools and the SWISS-MODEL database for protein modeling^2^. Multiple sequence alignment was performed with the sequences of SDPs using DNAMAN. A phylogenetic tree was established via the neighbor-joining tree available in MEGA7.0.9, and the schematic diagram of the carrier was constructed using IBS 1.0.1 software.

### Oligopeptide Transporter Activity Assay

Minimal medium base [1:50 dilution of the stock solution (26 g L^–1^ KCl, 26 g L^–1^ MgSO_4_, 76 g L^–1^ KH_2_PO_4_, 50 mL L^–1^ trace elements solution, pH 6.5)] was supplemented with glucose to a final concentration of 1%. Growth assays were carried out in Petri dishes (40 mm × 8 mm) filled with 10 mL solid growth medium. Each plate contained protease inhibitors [2 mM AEBSF (Sigma), 5 mM Pepstatin A (Sigma) and 1 mM EDTA (Sigma)] and 2–3 mg of a defined oligopeptide [KLLG, GGFL, or LWMR (Qiangyao BioTech, Shanghai)] in 10 mL culture medium as the only available nitrogen source.

### Microscopic Observation

In order to observe fungal development structures, such as hook-like structures, coil–coil structures, or related structures, the corresponding strains were cultured in a PDA plate in which coverslips were obliquely inserted into the culture medium. The coverslips were removed from the plate and observed under a microscope (Nikon 80i, Japan) after the mycelia covered all of each coverslip.

For the subcellular localization, the corresponding strains were cultured in a PDA plate in which coverslips were obliquely inserted into the culture medium for 5 days and observed via fluorescence microscopy after the mycelia covered all of each coverslip. The coverslips were removed from the PDA plate, washed twice gently with double distilled water, and incubated with 100 μL DiO perchlorate (Yeasen Biology, Shanghai, China) for 2–20 min. The cell membrane dye DiO was used to stain the cell membrane. Green fluorescence from DiO and red fluorescence from DsRED2 were observed under an Olympus Xa21 microscope (Olympus, Tokyo, Japan).

### Growth Rate and Lifespan Measurements

Lifespan was assayed according to the method of Geydan et al. (2012) with slight modification. In brief, lifespan was measured in time (days) and in length (cm) of continuous growth using Petri dishes (150 mm × 25 mm) filled with 65 mL of CM medium, culture in 50°C. Each measurement was performed at least in duplicate for each medium. The Petri dishes were incubated under an angle of 30–45°. Growth was scored three times every 5 days. Explants from the rims of over-grown Petri dishes were transferred to new Petri dishes with fresh medium to allow continued growth, and this process was repeated until the growth rate declined significantly. Strains were classified as non-senescent when the growth rate did not decline and there were no morphological changes. According to Geydan et al. (2012), fast senescing cultures showed a decline in growth rate and morphological changes, which were often followed by complete cessation of growth.

### Measurement of Conidial Germination and Germ Tube Elongation

We investigated whether conidial growth was defective based on conidial production, the conidial germination rate, and bud tube elongation. First, conidial production was assessed by growing 5-mm mycelial plugs of wild-type and mutant strains separately on PDA plates at 50°C. After 5 days of culture, the conidia of the strains were washed with distilled water and counted. To measure the germination rate, 100 μL of the conidial suspension (10^4^ conidia/mL) was mixed with 100 μL sterilized distilled water, added to each well of a 96-well plate, and incubated at 50°C. Once per hour, the appropriate experimental group was placed on a microscope slide and observed using a Nikon YS100 microscope (Nikon, Japan). Germ tube elongation was measured using photography software. Images of conidia germination were collected in the 5th hour. Each assay was independently repeated three times for each strain.

### Measurement and Qualitative Detection of Endogenous H_2_O_2_

The hydrogen peroxide content was determined as previously described for plants (Cui et al., 2017). Hydrogen peroxide was extracted by homogenizing 3 g of mycelia from wild-type and mutant strains in 6 mL of cold acetone. The homogenate was centrifuged at 3,500 × *g* for 5 min at room temperature, and the resulting supernatant was designated as the sample extract. Thereafter, 0.1 mL of titanium reagent [5% (w/v) titanic sulfate in concentrated H_2_SO_4_] was added to 1 mL of the sample extract, followed by the addition of 0.2 mL of strong aqua ammoniac to precipitate the peroxide-titanium complex. The precipitated sample was centrifuged at 3,000 × *g* for 10 min at room temperature, the supernatant was discarded, and the precipitate was solubilized in 5 mL of 2M H_2_SO_4_. The absorbance of the samples was determined at 415 nm against a 2M H_2_SO_4_ blank. The hydrogen peroxide concentration in the samples was determined by comparing the absorbance against a standard curve of 0–5 μM titanium-H_2_O_2_ complex. This experiment was performed in triplicate and independently repeated three times for each strain.

Lucigenin (*N,N*′-dimethyl-9,9′-bisacridinium dinitrate) (Invitrogen, Cat#: L 6868) is a chemiluminescence probe that emits blue-green fluorescence when it reacts with a variety of reducing agents. The lucigenin signal was excited by an argon laser at a wavelength of 457 nm. The fluorescence was used to qualitatively measure changes in the H_2_O_2_ concentration. Hyphae used in this experiment were treated with 0.01M PBS for 2 h before incubation with 10 μM lucigenin. Confocal images were collected using an Olympus Xa21 microscope (Olympus, Tokyo, Japan).

### Nitrogen Source Utilization Test

Nitrogen source utilization tests were conducted to determine the functions of TlSDP, CtSDP1, CtSDP2, and CtSDP3. Peptone, glutamic acid, reduced glutathione (GSH), tripeptide (KLG), and tetrapeptide (GGFL) were added separately to minimal medium [2 g (NH_4_)_2_SO_4_, 0.2 g MgSO_4_, 0.01 g CaCl_2_, 0.005 g FeSO_4_, 0.002 g MnCl_2_, 10.5 g K_2_HPO_4_, and 4.5 g KH_2_PO_4_ in 1000 mL distilled water, pH 7.5] replace the original nitrogen source ((NH_4_)_2_SO_4_) in the MM medium. Next, 5-mm mycelial plugs of the wild-type and mutant strains were cultured on the medium described above at 50°C for 5 days. The colonies were observed every day, and the colony diameter was measured after 5 days. Each sample was tested in three technical replicates. The experiment was repeated three times.

### Quantitative Real-Time PCR (qRT-PCR)

The conidia suspension was incubated in CM medium at 50°C for 4 days in a rotatory shaker (180 rpm) or left stationary. Mycelia were collected by filtration, and TRIzol (TaKaRa, Dalian) was used to extract total RNA from the samples. First-strand cDNA was synthesized using the EasyScript First-Stand cDNA Synthesis SuperMix kit (Trans). Subsequently, qRT-PCR was performed using the PCRmax Eco system. Relative quantification of each DNA sample was performed using a DNA binding dye (TB Green remix Ex Taq II, TaKaRa). The thermal program was as follows: 50°C for 60 s and 95°C for 90 s, followed by 40 cycles of 95°C for 5 s, 60°C for 34 s, and 72°C for 30 s, with a final step at 95°C for 15 s, 60°C for 60 s, and 95°C for 15 s. Each sample was tested in three replicates in each experiment, and the *actin* gene was used as an internal standard. The relative quantification of each sample was calculated as follows: 2^–ΔCq^ (Cq = Cq_gene_-Cq_actin_). For the figures, the expression quantity of the wild-type group was defined as 1, and the relative values of the other samples were calculated accordingly. The primer sequences used for qRT-PCR are listed in the Supplementary Information.

### Statistical Analysis

All experiments were repeated at least three times. The mean ± standard deviation of the colony diameter, germination rate, and relative expression were determined using GraphPad Prism 7.00 software. Data were analyzed using InStat3. The threshold for statistical significance was *p* < 0.05.

## Results

### *T. lanuginosus* and *C. thermophilum* Inhabiting the Same Environment Reproduce in Distinct Patterns

**FIGURE 1:**
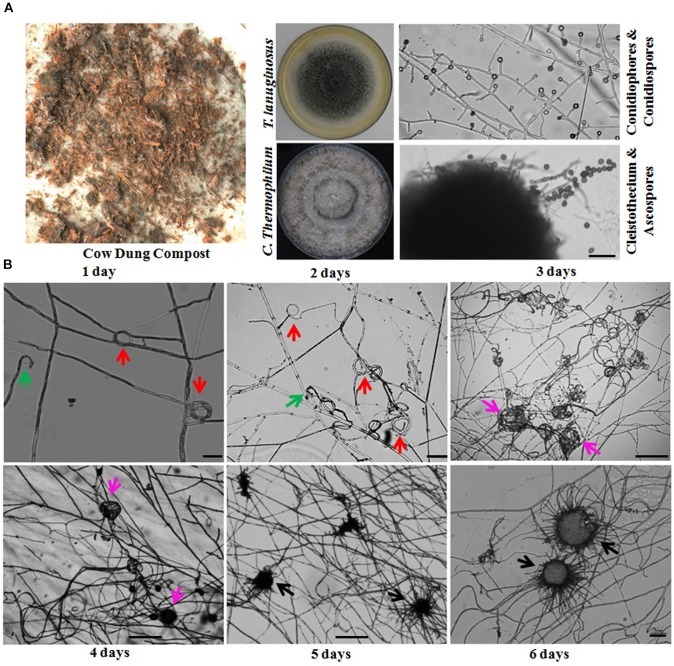
*Thermomyces lanuginosus* and *Chaetomium thermophilum* isolated from the same environment display different reproductive patterns. **(A)** A representative sample and the isolated *T. lanuginosus* and *C. thermophilum* strains. The cow dung sample was collected from Changchun, and the isolated strains were cultivated on CM plates under normal culture conditions for certain period; the *T. lanuginosus* conidiophores and conidiospores formed after 5 days, and the *C. thermophilum* cleistothecium and ascospore formed after 7 days. Bar = 20 μm. **(B)** Sexual lifecycle of *C. thermophilum.* The germinated ascospores were inoculated on CM media. The hook-like (green arrows), coil coil-like (red arrows), sclerotia-like (pink arrows), and immature cleistothecium structures (black arrows) formed at the apex of the hyphae on the different day. Bar = 50 μm.

Thermophilic molds are distributed ubiquitously and thrive in a variety of natural habitats, including soils, composts, wood chip piles, nesting materials of birds and other animals, and municipal refuse (Singh et al., 2016). In our laboratory, a large number of thermophilic fungi have been isolated and identified from cow dung, straw or compost piles in northeastern China since 2006. During our investigations, we found that sexual reproduction in *C. thermophilum* and asexual reproduction in *T. lanuginosus* were often found in the same samples (Figure 1A and Table 1). *T. lanuginosus* is classified as a Deuteromycete, which is defined as unicellular or septate with asexual reproduction by the formation of aleurioconidia (Hauser et al., 2000). Initially the colonies appear white and grow rapidly, reaching 9 cm in diameter at 50°C within 5 days, after which turn gray or greenish-gray beginning from the center of the colony. *C. thermophilum* is a thermotolerant, floccose fungus that produces typical concentric rings of growth. The ascomata of *C. thermophilum* are dark brown, superficial, globose, subglobose, or ovate. The peridium is brown, of textura angularis or irregularis. The sexual ascospores (Figure 1A) appear globose or subglobose.

**TABLE 1:** Investigation of the coexistence of *Tl* and *Ct* in a same sample.

	**Samples**				
**Year**	**Total**	**With *Tl* but without *Ct***	**With *Ct* but without *Tl***	**With both *Tl* & *Ct***
2006	319	49	34	211
2007	285	17	23	187
2008	196	14	9	164
2009	343	45	28	240
2010	302	37	21	222
2011	346	51	32	242
2012	425	55	46	301
2013	344	42	28	253
2014	202	11	9	170
2015	251	15	16	199
2016	235	18	13	181
2017	420	40	49	312
2018	314	38	25	231

**Tl*, *T. lanuginosus; Ct*, C. *thermophilum*; total samples (samples with both *Tl* and *Ct*) + (samples without both *Tl* and *Ct*) + (samples with *Tl* but without *Ct*) + (samples with *Ct* but without *Tl*).*

The life cycle of the thermophilic fungus *T. lanuginosus* is comprised of a vegetative mycelium growth stage and a conidial reproductive stage. When *T. lanuginosus* conidia are inoculated on CM plates under normal culture conditions (pH 7.0, 50–55°C, in darkness), the conidia immediately germinate and enter the mycelia growth stage. After 2 days of vegetative growth, *T. lanuginosus* enter the reproductive stage and subsequently produce conidia. On the fourth or fifth day, a large number of mature conidia are formed, which can germinate and enter the next life cycle (Supplementary Figure S2). In contrast to *T. lanuginosus*, the life cycle of *C. thermophilum* is extremely complex. It must go through a stage of morphogenesis and then produce sexual ascospores. As the starting point of development, germinated ascospores were prepared in advance and inoculated on CM media. After 1 day of growth and development, hook-shaped or coil–coil structures formed at the apex of the hyphae, which represented the initiation of sexual reproduction. Over the next 5 days, the vegetative mycelia gradually aggregated into spherical structures. Next, the related hyphal cells differentiated into sterile and fertile hyphal cells. Finally, the fruiting mycelium, containing both sterile and fertile hyphae, matured into the ascocarp (Figure 1B). As the ascospores mature and are released, a sexual reproductive cycle is completed.

### Four *isp4/SDP* Homologs and Their Expression Profiles

Based on the isp4 protein sequence reported by Sato et al. (1994), we performed a Blast-p search of the genomes of *T. lanuginosus* and *C. thermophilum*^1^. The four genes identified by the Blast-p search were designated *TlSDP, CtSDP1, CtSDP2*, and *CtSDP3.*

**FIGURE 2:**
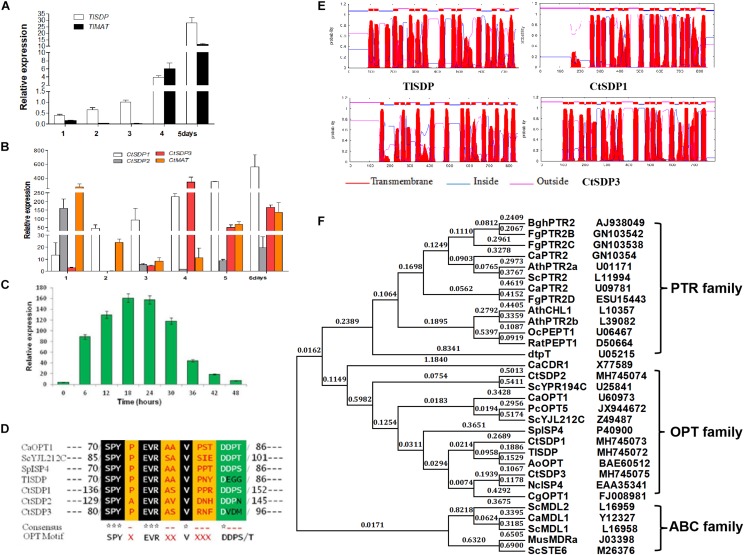
The *isp4/SDP* homologs in *T. lanuginosus* and *C. thermophilum*. **(A)** The expression pattern of *TlSDP* and *TlMAT* over 5 days. **(B)** The expression patterns of *CtSDP1, CtSDP2*, and *CtSDP3* over 6 days. **(C)** The expression pattern of *CtSDP2* over 48 h. **(D)** The single *isp4* homolog in *T. lanuginosus* (TlSDP) and the three *isp4* homologs in *C. thermophilum* (CtSDP1, CtSDP2, CtSDP3) contain the consensus sequence SPYxEVRxxVxxxDDP typical of OPT*s*. **(E)** TMHMM posterior probabilities for WEBSEQUENCE (on-line analysis). The analysis predicted 13–18 transmembrane-spanning domains, represented as red peaks. **(F)** Phylogenetic tree of oligopeptide transporter protein (OPT) sequences from various species. The full protein sequences from different eukaryotic organisms were analyzed, which revealed three major groups. The four *isp4/SDPs* homologs in this study grouped into the OPT clade.

To measure the expression levels of the identified genes, samples for mRNA extraction were collected at different stages according to the time course of growth and development (1, 2, 3, 4, and 5 days). By using qRT-PCR analysis, *TlSDP* expression was found to increase from the vegetative growth stage to the conidial formation and maturation stage (Figure 2A). *Tlmat*, a mating type determination gene that is essential for sexual reproduction in sexual fungi, appeared to be up-regulated during fungal development (Figure 2A), suggesting the involvement of *TlSDP* and *Tlmat* in the growth and development of *T. lanuginosus*.

Like *TlSDP*, *CtSDP1* expression was continuously elevated from the vegetative growth stage to the ascospores formation and maturation stage (Figure 2B). However, *CtSDP3* expression appeared to remain at a low level for the first 3 days but reached a much higher level on the fourth day, after which it decreased, but remained relatively high (Figure 2B). Surprisingly, *CtSDP2* expression was highest on the first day. On the second day, *CtSDP2* expression suddenly dropped from 160% to less than 5%; over the next 5 days, the relative expression level of *CtSDP2* remained at a level of less than 30%, although its expression level increased slightly beginning on the third day (Figure 2B). Considering the complexity of sexual morphogenesis in *C. thermophilum*, the distinct expression patterns of the three *CtSDPs* may reflect their different roles in sexual reproduction.

In order to obtain a more detailed expression profile of *CtSDP2*, fungal samples were collected every 6 h during the initial 48-h period. In ascospores that had just germinated, the expression level of *CtSDP2* was very low. *CtSDP2* expression gradually increased with germ tube elongation and hyphal growth. The expression level was highest from 18 to 24 h, after which it gradually decreased and reached its lowest level at 48 h (Figure 2C). Given the large number of hook-like structures at the top of the hyphae formed at this stage, *CtSDP2* is likely to be a relatively important factor during sexual morphogenesis.

Next, the structures of the proteins predicted to be encoded by *TlSDP, CtSDP1, CtSDP2*, and *CtSDP3* were analyzed using online tools. The corresponding predicted proteins (TlSDP and CtSDP1–CtSDP3) contain the consensus sequence SPYxEVRxxVxxxDDP (Figure 2D), which has been found in all previously studied OPTs (Lubkowitz et al., 1997, 1998). All four proteins contained 13–18 transmembrane-spanning domains^3^ and were most likely to be localized in the plasma membrane (Figure 2E and Supplementary Figure S3A). The phylogenetic analysis of SDP proteins was performed by the neighbor joining method (Rogaeva et al., 1999) with proteins from other fungi and plants (Supplementary Table S3B). The phylogenetic tree showed two major clades among the selected proteins. One clade contained members of the ABC family, whereas the other clade was further divided into two sub-branches containing the PTR and OPT families (Figure 2F). In the OPT family, CtSDP1 and CtSDP3 possessed close evolutionary relationships to TlSDP, but not to CtSDP2 (Supplementary Figure S3B). These analyses clearly demonstrate that the four SDPs belong to the OPT family and that CtSDP2 differs from the other three proteins.

### *CtSDP1–3* Play Different Roles in *TlSDP* Deletion Strain (Δ*TlSDP*)

**FIGURE 3:**
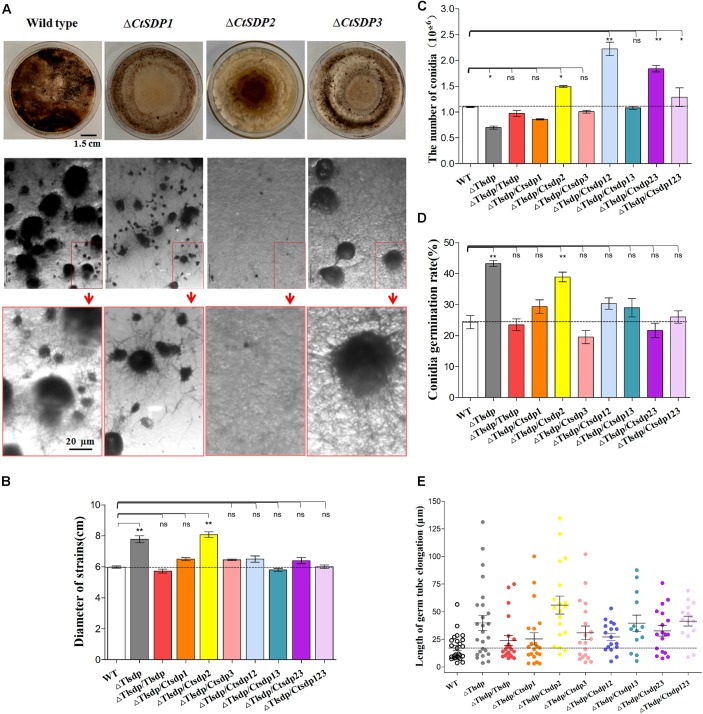
Biological roles of *isp4/SDP* genes in *T. lanuginosus* and *C. thermophilum.* **(A)** Colony morphologies of single-gene deletion mutants (Δ*CtSDP1*, Δ*CtSDP2*, and Δ*CtSDP3*) and the wild-type strains grown on CM plates at 50°C for 7 days (the first row); the representative microphotographic positions were selected randomly from the colonies of single-gene deletion mutants. The black spheroids represent sexual structures (the second row); the enlarged microphotographic pictures of colonies are shown in red rectangle boxes (the third row). Comparisons of **(B**) colony growth, **(C)** conidial production, **(D)** conidial germination rate, and **(E)** germ tube elongation between the Δ*TlSDP*, wild-type, and complemented strains (single, double, or triple expression of the three *CtSDPs*, based on the Δ*TlSDP* strains), grown on CM media at 50°C for 5 h. Error bars represent standard deviation. Significant differences between the wild-type strains and the two mutant strains are indicated as: ^∗^*p* < 0.05; ^∗∗^*p* < 0.01; ^∗∗∗^*p* < 0.001; or *ns*, not significant.

First, we constructed single gene deletion mutants (Δ*TlSDP*, Δ*CtSDP1*, Δ*CtSDP2*, and Δ*CtSDP3*) to verify that these genes function in their own strain (Supplementary Figures S1A, S6). In comparison with the *T. lanuginosus* wild-type strain, Δ*TlSDP* exhibited defective conidia, increased growth rate, and bud tube elongation (Figures 3B–E). The phenotype was more complex in the *C. thermophilum* single gene deletion mutants. In CM media, there was no significant change in colony growth among the deletion mutant strains, but significant alteration occurred at sexual morphogenesis (Figure 3A). Δ*CtSDP2* loss resulted in no sexual structures and no formation of the cleistothecium or ascospores (Figure 3A), confirming the requirement of *CtSDP2* for sexual structure initiation. In the Δ*CtSDP1* and Δ*CtSDP3* mutants, the cleistothecium and ascospores were produced, but the Δ*CtSDP1* mutant appeared to show distorted and retarded cleistothecium formation. The Δ*CtSDP3* mutant produced a smaller number of cleistothecia in comparison with the wild-type strain (Figure 3A). Interestingly, in the three mutant strains, loss of a single gene did not affect the expression of the other two genes (Supplementary Figure S4A), implying no mutual regulation among them.

To further verify the functional relationship of the four *SDP* genes, three *CtSDP* genes were constructed and transformed into the Δ*TlSDP* mutant strain (single, double, and triple transformation) (Supplementary Figures S1B–D). These strains were subjected to assessments of colony growth, conidial production and germination, and germ tube elongation. In comparison with the wild-type (*T. lanuginosus*) and Δ*TlSDP/TlSDP* strains, all complemented strains, with the exception of Δ*TlSDP/CtSDP2*, produced colonies of a similar size (Figure 3B and Supplementary Figure S4B). Interestingly, only Δ*TlSDP/CtSDP2* was not able to restore the growth phenotype of Δ*TlSDP* (Figure 3B), implying that *CtSDP1* and *CtSDP3*, but not *CtSDP2*, play the same role as *TlSDP* in growth.

With regard to sporulation, all SDP genes restored the defective conidia production of the ΔTlSDP strain (Figure 3C). Furthermore, sporulation of Δ*TlSDP/CtSDP2, ΔTlSDP/CtSDP12*, Δ*TlSDP/CtSDP23*, and Δ*TlSDP/CtSDP123* was higher or significantly higher than that of the wild-type strain during growth on PDA or CM media plates for 4 days (Figure 3C), further indicating the importance of *CtSDP2* in sporogenesis.

Conidial vitality was investigated by measuring the conidial germination rate and assessing germ tube elongation. The germination rate of the Δ*TlSDP*/*CtSDP2* strains was significantly greater than that of the wild-type strain (Figure 3D). In addition, assessment of germ tube elongation showed that the germinated spores continued to grow and develop (Figure 3E). The biological activities of the complemented strains suggested that the four tested genes are involved in growth, as well as the formation and development of conidia. Moreover, *CtSDPs*, especially *CtSDP2*, may play dual roles in sexual and asexual reproduction.

### Subcellular Location and Oligopeptide Transporter Activity of Four *SDPs* Genes

**FIGURE 4:**
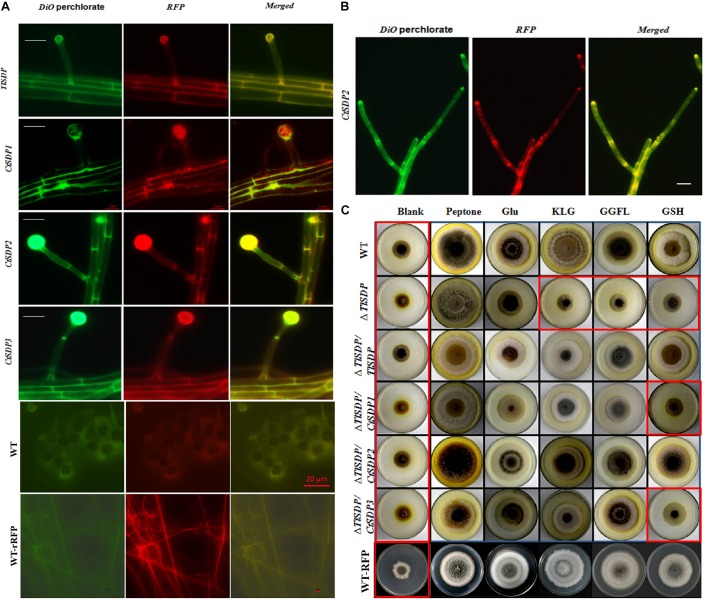
The OPT activity and subcellular locations of the four isp4/SDP proteins. **(A)** Intracellular localization of four SDP proteins. In the merged image, DiO perchlorate (green fluorescence) colocalized with DsRED (red fluorescence), appearing as yellow areas. Merged, green fluorescence overlapped with red fluorescence of DsRED. Images were acquired after 36 h of growth in CM medium. Bar = 10 μm. **(B)** CtSDP2/DsRED accumulated at the top, branch, and septum of the growth hyphae. Bar = 10 μm. **(C)** Biological analysis of OPT activity in a series of complemented strains expressing the four *SDP* genes. Growth experiments were performed on MM media lacking a nitrogen source (blank) and MM media supplemented with peptone, glutamic acid, GSH, tripeptide (KLG), or tetrapeptide (GGFL) as the sole nitrogen resource with all the tested strains. Strains in red rectangular boxes are inhibited in growth (thus, no OPT activity).

In order to verify whether the four SDPs has OPT activity, experiments assessing protein subcellular localization and nitrogen source utilization were performed. First, four DsRED-tagged *SDP* complementation strains based on Δ*TlSDP* were constructed. In these strains, every *SDP* gene was fused with *DsRED*, which was mainly used to determine the subcellular location of the fused protein (Supplementary Figure S1B). After 60 h of cultivation, DsRED red fluorescence co-localized with DiO green fluorescence on the hyphal cell membrane, septa and conidial surface in all four complementation samples, confirming that the four tested genes are localized on the cell membrane (Figure 4A). In addition, in the *CtSDP2*/DsRED fusion strains, the merged fluorescence signal was markedly accumulated at the top, branch, and septum of the growth hyphae (Figure 4B), implying that *CtSDP2* is associated with fungal growth and development.

The coding proteins of *Isp4* and its homologs are OPTs (Sato et al., 1994), and the four SDP proteins identified in this study also contain OPT family special sequence SPYxEVRxxVxxxDDP. To verify the transporter ability of the SDPs, growth experiments on MM media were carried out with different nitrogen sources. *T. lanuginosus* wild-type, Δ*TlSDP/TlSDP* and Δ*TlSDP* strains grew poorly on MM media lacking a nitrogen source, but the wild-type and Δ*TlSDP/TlSDP* strains grew well when synthesized tripeptides (KLG) or tetrapeptides (GGFL) were added as the sole nitrogen resource (Figure 4C). This experiment confirmed that TlSDP is an OPT, at least for the oligopeptide used in this research.

The growth of Δ*TlSDP/CtSDP1, ΔTlSDP/CtSDP2*, and Δ*TlSDP/CtSDP3* was assessed on nitrogen-deficient media or media supplemented with a nitrogen source such as tetrapeptides. Additionally, protease inhibitors were added to limit degradation of oligopeptides by secreted proteases. Among several oligopeptides tested, the tetrapeptide GGFL revealed the most obvious growth phenotype. On nitrogen-deficient media supplemented with GGFL, Δ*TlSDP* growth was extremely retarded; however, all the three complemented strains, as well as Δ*TlSDP/TlSDP*, grew normally (Figure 4C). Unexpectedly, when GSH was used as the sole nitrogen source, only *TlSDP* and *CtSDP2* were able to restore the defective growth of Δ*TlSDP* (Figure 4C), suggesting that TlSDP and CtSDP2 are involved in glutathione transportation.

### Life Span Is Extended in Δ*CtSDP2* and Shortened in Δ*TlSDP/CtSDP2*

**FIGURE 5:**
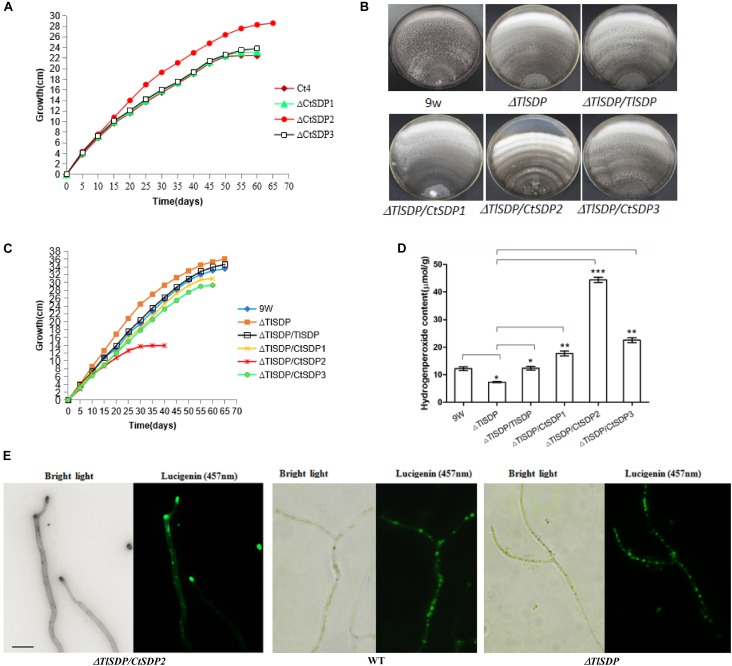
Lifespan is extended in Δ*CtSDP2* and shortened in Δ*TlSDP/CtSDP2.* **(A)** Δ*CtSDP2* exhibited constant growth for greater than 65 days. When compared to the Δ*CtSDP2* strain, the Δ*CtSDP1, ΔCtSDP3*, and S4 wild-type strains exhibited slowed growth phases, followed by a halt in growth at approximately less than 58 days. Growth curves were established from 30 subcultures of six independent spores for each genotype (S4, Δ*CtSDP1*, Δ*CtSDP2*, and Δ*CtSDP3*). **(B)** The mycelial phenotype of Δ*TlSDP*, Δ*TlSDP/TlSDP*, Δ*TlSDP/CtSDP1*, Δ*TlSDP/CtSDP2*, and Δ*TlSDP/CtSDP3* inoculated on CM medium at 20 days. **(C)** The growth curves of Δ*TlSDP*, Δ*TlSDP/TlSDP*, and Δ*TlSDP/CtSDP1-3* inoculated on CM medium. The Δ*TlSDP/CtSDP2* strains displayed a slow growth phase, followed by a halt in the growth phase at approximately less than 35 days. Other tested strains exhibited constant growth for more than 60–65 days. Growth curves were established from 30 subcultures of six independent spores for each genotype. **(D)** Hydrogen peroxide (H_2_O_2_) content of the Δ*TlSDP*, Δ*TlSDP/TlSDP*, Δ*TlSDP/CtSDP1*, Δ*TlSDP/CtSDP2*, and Δ*TlSDP/CtSDP3* strains. Significant differences between the wild-type strains and the two mutant strains are indicated as: ^∗^*p* < 0.05; ^∗∗^*p* < 0.01; ^∗∗∗^*p* < 0.001; or *ns*, not significant. Error bars represent standard deviation. **(E)** The Δ*TlSDP/CtSDP2* strains accumulate more H_2_O_2_ at the top of their hyphae. The light (left) and fluorescence confocal (right) microscope observations of a Δ*TlSDP/CtSDP2* hypha. Bar = 10 μm.

During incubation and storage with PDA or CM at room temperature or 50°C, almost all isolated *C. thermophilum* strains in our lab were susceptible to aging and death in fewer than 60 days (Supplementary Table S4). Life span analysis of the *C. thermophilum* wild-type S4 strain and the three Δ*CtSDP* strains was carried out according to Geydan et al. (2012) and Cui et al. (2017). In the third culture cycle, growth of the S4, Δ*CtSDP1*, and Δ*CtSDP3* strains stopped on the 15^*th*^–18^*th*^ day, meaning the life span of these fungi was less than 58 days (Figure 5A). However, the Δ*CtSDP2* strain has an extended lifespan of more than 65 days (Figure 5A). Thus, we speculate that *CtSDP2* has a negative effect on fungal lifespan.

Next, we performed lifespan measurement for the *T. lanuginosus* wild-type strain and five mutants (Δ*TlSDP/TlSDP*, Δ*TlSDP*, Δ*TlSDP/CtSDP1*, Δ*TlSDP/CtSDP2*, and Δ*TlSDP/CtSDP3*). In the first culture cycle, the Δ*TlSDP/CtSDP2* strains showed obvious mycelial growth reduction and concentric undulate hyphae (Figure 5B). In the second 20-day culture cycle, Δ*TlSDP/CtSDP2* growth stopped on the 15^*th*^ day, indicating that the maximum lifespan of the Δ*TlSDP/CtSDP2* strain was approximately 35 days (Figure 5C). The growth of the Δ*TlSDP* strains on CM was linear at 65 days, indicating that its lifespan was greater than 65 days. The lifespans of the Δ*TlSDP/TlSDP*, Δ*TlSDP/CtSDP1*, and Δ*TlSDP/CtSDP3* strains ranged from 60 to 65 days (Figure 5C). These results show that *CtSDP2*, which has a negative effect on the lifespan of *C. thermophilum*, plays a similar role in heterologous species.

Reactive oxygen species (ROS) are associated with reduced longevity (Bianchi and Falcioni, 2016; Nita and Grzybowski, 2016), and ROS production has been regarded as the primary cause of biological aging (Harman, 1972; Bianchi and Falcioni, 2016; Nita and Grzybowski, 2016). Thus, we compared the hydrogen peroxide content of the Δ*TlSDP* and Δ*TlSDP/CtSDP2* strains. The hydrogen peroxide content of the Δ*TlSDP/CtSDP2* strain was significantly higher than that of the Δ*TlSDP* strain (Figure 5D). In addition, accumulation of hydrogen peroxide at the tops of hyphae was observed in the Δ*TlSDP/CtSDP2* strain (Figure 5E), but not in the Δ*TlSDP* strain, partially explaining the relationship between longevity and *CtSDP2* expression.

### The 72-nt Insertion Fragment (72INS) of *CtSDP2* and Its Role in Sexual Development

**FIGURE 6:**
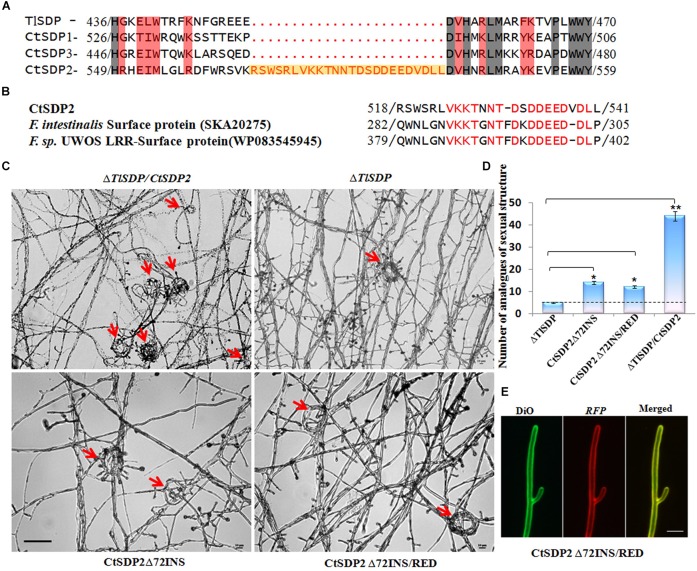
The 72-nt insertion fragment (72INS) of CtSDP2 and its role in sexual development. **(A)** Alignments of the predicted amino acid sequences of TlSDP, CtSDP1, CtSDP2, and CtSDP3. An extra oligopeptide fragment (72INS) consisting of 24 amino acid residues between 518 (Arg) and 541 (Leu) is highlighted in the CtSDP2 protein. **(B)** Amino acid sequences similar to the 72INS (24 amino acid residues) of CtSDP2. Identical amino acid residues are indicated by red letters in CtSDP2, F. intestinalis surface protein (SKA20275), and F. sp. UWOS LRR surface protein (WP083545945). **(C)** Phenotypic comparison of the Δ*TlSDP/CtSDP2*, Δ*TlSDP*, *CtSDP2Δ72INS*, and *CtSDP2Δ72INS/RED* strains. Phenotypes of the tested strains grown on solid CM for 4 days were observed. The analogs of the sexual structure, such as coil coil-like structures (red arrows), were observed. Bar = 50 μm. **(D)** The average number of analogs of sexual structures of the tested strains observed in different microscope vision fields. Significant differences between the relative strains are indicated as: ^∗^*p* < 0.05; ^∗∗^*p* < 0.01; ^∗∗∗^*p* < 0.001; or *ns*, not significant. **(E)** Intracellular localization of the CtSDP2Δ72INS/RED protein. In the merged image, DiO perchlorate (green fluorescence) colocalized with DsRED (red fluorescence), appearing as yellow areas. CtSDP2Δ72INS/RED proteins no longer accumulated on the top of the hyphae, and the hyphal septa at the apex of the hyphae or near the hyphal branches were barely observed. Images were acquired after 36 h of growth in CM medium. Bar = 10 μm.

To determine why the role of *CtSDP2* differs from the four *SDPs*, their protein sequences were aligned (Supplementary Figure S5). An extra oligopeptide fragment consisting of 24 amino acid residues from Arg518 to Leu541 (Figure 6A) was found in CtSDP2. This 24-amino acid oligopeptide fragment is encoded by 72 nucleotides (nt) that appear to be inserted into the original sequence of *CtSDP2* (Supplementary Figure S7), and it was designated 72INS. A website Blast search indicated that 72INS might be associated with surface proteins and leucine rich repeat proteins (Figure 6B).

In order to explore the function of 72INS, we constructed two 72INS-deletion vectors based on the pKD7-RED-CtSDP2 vectors (Supplementary Figures S1B,C), in which 72INS was deleted using La-TAQ PCR amplification (Supplementary Table S1). The transformant containing *CtSDP2Δ72INS* (*CtSDP2* lacking *72INS*) was obtained and compared with strains containing the corresponding full-length sequences in the Δ*TlSDP* strain. No significant differences were found between the tested strains in terms of colony phenotype, growth rate, sporogenesis, or OPT absorption. However, the number of hook-shaped or coil–coil structures was sharply reduced in the 72INS deletion strains (Figures 6C,D). Therefore, 72INS may contribute to the initiation of sexual morphogenesis, given that *CtSDP2* is required for sexual structure formation in *C. thermophilum* (Figure 3A) and the Δ*TlSDP/CtSDP2* strain formed more analogs of sexual structures (hook-like or coil–coil structures) than did the Δ*TlSDP/CtSDP2Δ72INS* strain (Figure 6C). The DsRED-tagged proteins did not accumulate at the tops of the hyphae. In addition, hyphal septa at the hyphal apex or near the hyphal branches were hardly observed (Figure 6E). These results reflect the involvement of 72INS in CtSDP2 accumulation at the tops of hyphae and septum formation.

## Discussion

Peptide transport across membranes is accomplished by diverse organisms. At present, three families of transporters have been intensively studied: the ABC (ATP binding cassette) family, the PTR (peptide transport) family, and the OPT family (Stacey et al., 2002). The ABC superfamily transports a large variety of substances (not restricted to peptides) (Payne and Smith, 1994). The PTR family transports amino acids, dipeptides, tripeptides, and nitrate (Stacey et al., 2002). In contrast to the ABC and PTR families, OPTs predominantly transport tetrapeptides and pentapeptides (Lubkowitz et al., 1997, 1998). In this study, the unique *TlSDP* gene of *T. lanuginosus* was cloned and biologically analyzed through creating the *TlSDP* gene deletion strains and complementation strains. As to the three *SDPs* in *C. thermophilum*, we successfully obtained three single gene deletion mutants (Δ*CtSDP1-3*), and at least three independent transformation colonies for each *CtSDP* mutants were isolated and checked for the defects. Unfortunately, we did not get the corresponding complementary strains. (We have tried many times, and each time we screened nearly 1000 candidate transformants, but they were identified as not having complementary genes.) Therefore, based on the Δ*TlSDP* strains, we transformed each of *CtSDP1-3* in the *T. lanuginosus* system, which we have successfully used in previous study. The four SDPs showed tetrapeptide transport activity according to the results of complementary growth experiments under nitrogen deficiency conditions (Figure 4C), confirming their assignment to the OPT family (Figure 2F). In addition, TlSDP and CtSDP2, in contrast with the other two SDPs, possessed the ability to transport glutathione, suggesting that TlSDP and CtSDP2 are glutathione transporter like Hgt1p (Opt1p) (Bourbouloux et al., 2000) and HGT1 (Dworeck et al., 2009).

Peptide transport systems are involved in multiple cellular processes, such as yeast mating and fungal pathogenicity (Kuchler et al., 1989; McGrath and Varshavsky, 1989; Stacey et al., 2002; Chague et al., 2009). The *Blumeria graminis PTR2* gene was reported to be expressed in infection structures (Droce et al., 2015). In addition, the *Fusarium graminearum PTR2* gene is associated with perithecium development and conidial production (Droce et al., 2017). Interestingly, our *SDPs* seemed to share some similarities with *PTR2* regarding sexual development-related biological functions.

The *TlSDP* is the only isp4 homolog in *T*. *lanuginosus.* Along with the three *CtSDP* genes, *TlSDP* was shown to be an OPT member based on growth experiments using complementary transformants (Figure 4C), The presence of fewer OPTs implied that an alternative system is required for nitrogen source absorption in *T. lanuginosus.* In the growth assay for OPT identification, differences in colony growth between the *SDP* deletion strains and the complementary strains were observed only when protease inhibitors were added to nitrogen-deficient media supplemented with polypeptides (data not shown), indicating that a powerful protease-dependent secretory degradation system exists in *T. lanuginosus*. Surely, other peptide transport systems, including members of the PTR families, may be involved in the utilization of nitrogen resources, because multiple sequences similar to those of PTR members were found in the genomes of *T. lanuginosus* and *C. thermophilum*^4^^,^^5^. With regard to nitrogen uptake, the important, but inessential, OPT activity of CtSDPs does not fully explain their biological functions in sexual development, especially the initial role of *CtSDP2* in sexual reproduction. Thus, the molecular mechanism of *SDPs* in sexual reproduction must be further studied.

In this study, we have created a series of mutants and complementary transformant strains, which provide rich materials for functional analyses of different *SDPs*. Each of the three deletion mutants of *CtSDP1–CtSDP3* contains the other two genes; therefore, the phenotype of the single-gene deletion strains did not entirely reflect the effect of the deleted gene. However, the requirement of *CtSDP2* for sexual morphogenesis was confirmed, because no sexual structures were observed in the Δ*CtSDP2* strain. Although we failed to obtain double- or triple-deletion *CtSDP* mutants, the Δ*TlSDP* strain served as an important genetic tool that enabled us to study *CtSDPs*. In addition, the reduced conidial production and defective asexual reproduction of the Δ*TlSDP* strain made it suitable for experiments assessing sporulation and sexual reproduction. Biological analyses of the functions of *CtSDP1*, *CtSDP2*, and *CtSDP3* were performed using the Δ*TlSDP* system. The observed enhancement of conidial production in the Δ*TlSDP/CtSDP* strain indicates the dual role of *CtSDPs.*

*CtSDP2* expression tended to reduce the longevity of fungus (Figures 5A,B). This effect was probably associated with energy metabolism, given the enormous energy requirements for growth and development (Cui et al., 2017). H_2_O_2_ and other ROS induce aging, and the production of free ROS, such as H_2_O_2_, in cells is the primary cause for biological aging (Harman, 1972; Falcioni and Bianchi, 2016; Nita and Grzybowski, 2016). In comparison with the Δ*TlSDP* strains, Δ*TlSDP/CtSDP2* accumulated more H_2_O_2_ (Figure 5D), and the lifespan of the Δ*TlSDP/CtSDP2* strain was much shorter than those of the Δ*TlSDP* strains (Figure 5C).

Insertion and deletion events cannot be accurately identified if ancestral sequence information is deficient. Human genetic evidence suggests that insertion and deletion are major sources of gene defects (Smith et al., 1997; Rogaeva et al., 1999; Mcgovern et al., 2005; Volfovsky et al., 2009). In *Bacillus subtilis*, *Escherichia coli*, and *Saccharomyces cerevisiae*, insertions and deletions are not randomly distributed and are likely to occur more often in essential proteins and those that are highly connected, indicating a possible role of sequence insertions and deletions in the regulation and modification of protein–protein interactions (Chan et al., 2007). In our research, a 24-amino acid oligopeptide fragment (encoded by 72 nt DNA sequence) was only found in CtSDP2, suggesting that a 72-nt DNA fragment was inserted into the original sequence of *CtSDP2*, if the other three *SDPs* are ancestral sequence. Sequences similar to 72INS are contained in some surface proteins and are likely to play important roles in initial adherence to mucosal tissue, as well as in long-term survival of the pathogen on mucosal surfaces (de Miguel et al., 2010). Thus, *CtSDP2* gained extra functions in comparison with the other three *SDPs* (Figure 6A).

Insertion and deletion are evolutionary changes in the sequence length of DNA and protein molecules (Britten et al., 2003; Tao et al., 2007). To our knowledge, the functions of insertions or deletions are not well-understood. An in-depth study of 72INS will provide a valuable reference for us to carry out functional insertion sequence research. This study is the first to link the expression of *SDP/isp4* genes to their sexual differentiation functions in *C. thermophilum* and *T. lanuginosus*. In particularly, we confirmed that *CtSDP2* harbors a unique functional 72INS that is required for sexual morphogenesis.

## Data Availability Statement

The datasets generated for this study can be found in NCBI, accession numbers MH745073, MH745074, MH745075, and MH745072.

## Author Contributions

S-HZ, X-LX, and YW conceptualized the study. X-LX, YW, Y-YS, G-MP, and S-HZ worked on the data curation. X-LX, YW, Y-YS, G-MP, L-NC, and S-HZ did the formal analysis. S-HZ and YW were responsible for the funding acquisition and the project administration. X-LX, YW, Y-YS, G-MP, L-NC, GW, and S-HZ carried out the investigation. X-LX, YW, Y-YS, and S-HZ worked on the methodology. S-HZ supervised the study and X-LX wrote, reviewed and edited the original draft.

## Conflict of Interest

The authors declare that the research was conducted in the absence of any commercial or financial relationships that could be construed as a potential conflict of interest.

## Footnotes

1 https://www.ncbi.nlm.nih.gov/assembly/GCA_000315935.1/

2 https://www.swissmodel.expasy.org/

3 http://www.cbs.dtu.dk/services/TMHMM/

4 http://fungi.ensembl.org

5 https://blast.ncbi.nlm.nih.gov/Blast.cgi
