# Supplementary material for: Genetic Analysis of Four Sexual Differentiation Process Proteins (isp4/SDPs) in Chaetomium thermophilum and Thermomyces lanuginosus Reveals Their Distinct Roles in Development
Source: Front Microbiol. 2020 Jan 6;10:2994. doi: 10.3389/fmicb.2019.02994 (PMC6956688; doi:10.3389/fmicb.2019.02994)

Supporting Information

Figures

Figure S1.Construction of gene deletion and complementation vectors. (A) Vectors for deletion of *TlSDP* and *CtSDPs*. LB, upstream flanking sequences of the *SDP* genes; RB, downstream flanking sequences of the *SDP* genes; *HYG*, hygromycin B phosphotransferase resistance gene. Genomic DNA from wild-type *T. lanuginosus* 9W and *C. thermophilum* S4 was used for amplification of the upstream and downstream flanking sequences. (B) Single-gene complementation vectors. The coding sequences of *TlSDP*, *CtSDP1*, *CtSDP2*, and *CtSDP3* were amplified from 9W and S4 genomic DNA. The first three sequences were inserted between the *XbalI* and *Sal*I sites, and the last was inserted between the *Bam*HI and *Sma*I sites of the pKD7-RED vector to generate the final complementation vectors, pKD7-RED-*TlSDP*, pKD7-RED-Ct*SDP1*, pKD7-RED-Ct*SDP2* and pKD7-RED-Ct*SDP3*. PH3, H3 promoter; DsRED2, red fluorescence marker; NEO, neomycin phosphotransferase gene. (C) The transition plasmids. The coding sequences of *CtSDP1*, *CtSDP2*, and *CtSDP3* were amplified from 9W and S4 cDNA; each of the digested fragments was ligated into the same double-digested pKAFCR80 vector, resulting in three transition plasmids, pKAFCR80-Ct*SDP1*, pKAFCR80-Ct*SDP2*, and pKAFCR80-Ct*SDP3*. 35S-P, cauliflower mosaic virus 35S promoter; NOS-T, nopaline synthase terminator. (D) Polygenic complementation vectors. The 35S-P-*CtSDP1*-NOS-T, 35S-P-*CtSDP2*-NOS-T, and 35S-P-*CtSDP3*-NOS-T fragments were double-digested with *Cla*I and *Pmac*I from the transition plasmids. The recovered 35S-P-*CtSDP1*-NOS-T fragment was ligated into the modified pKAFCR100 plasmid to generate the pKAFCR100-CtSDP1 vector. The 35S-P-CtSDP2-NOS-T fragment was then ligated into this vector to generate the pKAFCR100-*CtSDP1*-*CtSDP2* vector. Similarly, the 35S-P-*CtSDP3*-NOS-T fragment was ligated into the pKAFCR100-*CtSDP1*-*CtSDP2* vector and double-digested with *Cla*I and *Hap*I, resulting in the three-gene expression vector pKAFCR100-*CtSDP1*-*CtSDP2*-*CtSDP3*. 35-Ω-P, 35S promoter with an additional omega element translational enhancer; sGFP, synthetic green-fluorescent protein.

Figure S2. Development of *T. lanuginosus* from growth to reproduction. *T. lanuginosus* 9W strains were cultured on PDA plates in which coverslips were obliquely inserted into the culture medium near the strains (50°C, darkness) for 5 days.

The coverslips were removed from the plate and observed under a microscope every 24 hours.

Figure S3. Membrane topologies and phylogenetic analysis of the four isp4/SDP proteins. (A) Possible membrane topologies of the four proteins were predicted by hydropathy profiles (TMHMM posterior probabilities for WEBSEQUENCE). From this analysis, 13-18 transmembrane-spanning domains were predicted. (B) Phylogenetic tree of OPT from various species. The full protein sequences from different eukaryotic organisms were analyzed.

Figure S4. Expression patterns of the remaining two *CtSDP* genes in single-gene deletion strains of *C. thermophilum* and the colony phenotypes of complemented strains of *T. lanuginosus*. (A) Loss of a single *CtSDP* gene did not affect the expression profiles of the other two genes. (B) When compared to the wild-type and Δ*TlSDP*/*TlSDP* strains, all the complemented strains, except for Δ*TlSDP*/*CtSDP2*, grew to similar colony sizes.

FigureS5. The amino acids sequence alignment result between the four isp4/SDP proteins. The gray color represents four identical amino acids, the red represents three. The line under the sequences stand for the transmembrane-spanning domains.

FigureS6. The agarose gel electrophoresis for PCR validation of gene knockout and gene expression strains. (A) The PCR verification of *TlSDP,CtSDP1*,*CtSDP2* and *CtSDP3* single gene gene deletion strain. (B) The PCR verification of *TlSDP*, *CtSDP1*,*CtSDP2* and *CtSDP3* single and multigene expression strain. 9W: the *T. lanuginosus* wild type strain, Ct4: the *C. thermophilum* wild type strain, *TlSDP,CtSDP1,CtSDP2* and *CtSDP3*: the target gene, Hph ： Hygromycin amphotericin B， L+Hph: the fragment from upstream of the left border of the target gene to the middle of hygromycin gene. NPTII：neomycin phosphotransferase gene.

Figure S7. The cDNA sequence alignment result between the four *isp4/SDP* genes. The red color represents 72INS sequences.

Tables

Table S1. Nucleotide sequences of primers used in this study

Primer	Primer sequence	Restriction Enzyme
AtTlSDP-LB-F	5'-CTCGAGGAAGACTTTGGCAGTTGG-3'	XhoI
AtTlSDP-LB-R	5'-GAGCTCACATCGGTAGGAAGATTGG-3'	SacI
AtTlSDP-RB-F	5'-GGATCCTGCTTTCAACTCCTCTCG-3'	BamHI
AtTlSDP-RB-R	5'-AAGCTTGGCTGTTTAGACTTGGGT-3'	HindIII
AT-CtSDP1-LB-F	5'-GAAGATCT GGTTTCTGGATTCGTCTATG-3'	BglII
AT-CtSDP1-LB-R	5'-GGACTAGTTGGTGTTGTGATGGAGTC-3'	SpeI
AT-CtSDP1-F	5'-GAGGACGAGGATGAATACA-3'	
AT-CtSDP1-R	5'-CAGGACTGTCAGGGAATA-3'	
AT-CtSDP1-RB-F	5'-GCTCTAGATGAGTCATCGTTCCTTCC-3'	XbaI
AT-CtSDP1-RB-R	5'-CCAAGCTTTTGGACCAGGGTTACATC-3'	HindIII
AT-CtSDP2-LB-F1	5'-CGAGCTCCGTTTGGGCTTATCGTAG-3'	SacI
AT-CtSDP2-LB-R1	5'-GGGGTACCTATGAATGGTGGTAGGGC-3'	kpnI
AT-CtSDP2-F	5'-TAGATACCACCAACACCC-3'	
AT-CtSDP2-R	5'-GCTCCTAACAATGCTACAC-3'	
AT-CtSDP2-RB-F1	5'-GCTCTAGAGTCTTCGTGTTATCTGCC-3'	Xbal
AT-CtSDP2-RB-R1	5'-GCGTCGACCTTACTGATGTGGTGGGT-3'	SalI
AT-CtSDP3-LB-F4	5'-CCCTCGAGCACTTTGAGGCTTGGAAC-3'	XhoI
AT-CtSDP3-LB-R4	5'-CGGAATTCACTGTCTGGTGCGTTTGT-3'	EcoRI
AT-CtSDP3-F	5'-ATGTTGCTCTCCCTCAGA-3'	
AT-CtSDP3-R	5'-GAAATGATACTCCAGTCCTG-3'	
AT-CtSDP3-RB-F	5'-GCTCTAGACAGGTAGACAATAAGTGGAC-3'	XbaI
AT-CtSDP3-RB-R	5'-CCAAGCTTGGGAAGAGAGCATTCTGA-3'	HindIII
TlSDP-F	5'-TACATCGCACCCTTCCTCAG-3'	
TlSDP-R	5'-AACAGCATCATCGCCATCG-3'	
HYG-F	5'-GATGTAGGAGGGCGTGGATATGTCCT-3'	
HYG-R	5'-AACCCGCGGTCGGCATCTACTCTATTC-3'	
cTlSDP-F	5'-GCTCTAGAGCATGGAGGAGAAGATCAGC-3'	XbaI
cTlSDP-R	5'-GTCGACCCCGGCTGTCTCACCACT-3'	SalI
CtSDP1-F1	5'-GCTCTAGAGCATGGCTCATCCGTCGGGC-3'	XbaI
CtSDP1-R1	5'-GTCGACTTAAATCCTCCAACTATTCG-3'	SalI

CtSDP2-F1	5'-GCTCTAGAGCTCAATGGAACTCTCCGATGC-3'	XbaI
CtSDP2-R1	5'-GTCGACATGACAGGCGCGTCTGAGAA-3'	SalI
CtSDP3-F1	5'-CGGGATCC ATGGTTATGGCATTTCTC-3'	BamHI
CtSDP3-R1	5'-CCCCCGGG TCATGGCCAGCTGCTTGG-3'	SmaI
CtSDP1-F2	5'-GCTCTAGAATGGCTCATCCGTCGGGC-3'	XbaI
CtSDP1-R2	5'-GGGGTACCTTAAATCCTCCAACTATT-3'	KpnI
CtSDP2-F2	5'-CCCCCGGGATGACAGGCGCGTCTGAG-3'	SmaI
CtSDP2-R2	5'-CGAGCTCTCAATGGAACTCTCCG-3'	SacI
CtSDP3-F2	5'-GCTCTAGAATGGTTATGGCATTTCTC-3'	XbaI
CtSDP3-R2	5'-CCCTCGAGTCATGGCCAGCTGCTTGG-3'	XhoI
DsRED-P1	5'-AACCCGGGATGGCCTCCTCCGAGAACGTCATC-3'	
DsRED-P2	5'-AATCTAGACAGGAACAGGTGGTGGCG-3'	
NEO-SXF	5'-GCACTAGTGAGGTCAACACATCAATGC-3'	
NEO-SXR	5'-TTTCTGAGTCAGAAGAACTCGTCAAGAAGGCG-3'	
RT-TlSDP-F	5'-GGAGCGGAATACAATGTCAG-3'	
RT-TlSDP-R	5'-GCGGAACAGTCTTGAATCG-3'	
RT-CtSDP1-F	5'-GACCATTCAACTCAACCC-3'	
RT-CtSDP1-R	5'-ACTTCCTCATCATCCCAG-3'	
RT-CtSDP2-F	5'-TCCTTCATCAACGCCTTC-3'	
RT-CtSDP2-R	5'-GCGGTAAGACTTGTATCCACAC-3'	
RT-CtSDP3-F	5'-CTACGAGGACAAATGGCT-3'	
RT-CtSDP3-R	5'-GAAGAAGATGGTAACGCCT-3'	
Tlactin-F	5'-TCATCACCGTTGACCCTTTC-3'	
Tlactin-R	5'-ACGAGTCCTTCTGACCCATA-3'	
RT-Ctactin-F	5'-GAGGTTGCTGCTCTCGTT-3'	
RT-Ctactin-R	5'-CAGTCTTCTCCATGTCG-3'	
CtSDP2 -Δ72INS-F	5'-GATGTCCACAACCGCCTGATGCG-3'	
CtSDP2 -Δ72INS-R	5'-TTTCACGCTGCGCCAGAAGTCACG-3'	
NPT-F	5'-AGATGGATTGCACGCAGGTTCTCCG-3'	
NPT-R	5'-ACTCGTCAAGAAGGCGATAGAAGGCG-3'	

Note: LB, left border; RB, right border; HYG, hygromycin B phosphotransferase; DsRED; fluorescence of red color gene, NEO; neomycin phosphotransferase II gene; F, forward; R, reverse; SDP, Sexual differentiation process protein isp4; C, complete

sequence; RT, real time PCR; CtSDP2 -Δ72INS, 72 nt insertion fragment was deleted in *CtSDP2*. NPT-F/R: neomycin phosphotransferase gene.

Table S2. The deletion mutant or complemented strains and the constructed plasmids used for transformation in this study

Strains Created	Genotype	Plasmid Used
ΔTlSDP	*TlSDP-PXEH*	*TlSDP-PXEH*
Δ CtSDP1	*CtSDP1-PXEH*	*CtSDP1-PXEH*
Δ CtSDP2	*CtSDP2-PXEH*	*CtSDP2-PXEH*
Δ CtSDP3	*CtSDP3-PXEH*	*CtSDP3-PXEH*
ΔTlSDP/TlSDP	Δ*TlSDP::[TlSDP]*	*pKD-7- TlSDP*
ΔTlSDP/CtSDP1	Δ*TlSDP::[CtSDP1]*	*pKD-7- CtSDP1*
ΔTlSDP/CtSDP2	Δ*TlSDP::[CtSDP2]*	*pKD-7- CtSDP2*
ΔTlSDP/CtSDP3	Δ*TlSDP::[CtSDP3]*	*pKD-7- CtSDP3*
ΔTlSDP/CtSDP12	Δ*TlSDP::[CtSDP1]::[CtSDP2]*	*pKACR100-CtSDP12*
ΔTlSDP/CtSDP13	Δ*TlSDP::[CtSDP1]::[CtSDP3]*	*pKACR100-CtSDP13*
ΔTlSDP/CtSDP23	Δ*TlSDP::[CtSDP2]::[CtSDP3]*	*pKACR100-CtSDP23*
ΔTlSDP/CtSDP123	Δ*TlSDP::[CtSDP1]::[CtSDP2]:: [CtSDP2]*	*pKACR100-CtSDP123*
ΔTlSDP/ CtSDP2-Δ72INS	ΔTlSDP::[CtSDP2Δ72INS]	pKD-7- CtSDP2Δ72INS

Table S3. A list of the proteins used to generated the phylogenetic tree in Figure 2.

Protein	Source	Reference	Accession no.
AthCHL1	*A. thaliana*	Tsay et al. (1993)	L10357
AthPTR2a	*A. thaliana*	Steiner et al. (1994)	U01171
AthPTR2b	*A. thaliana*	Song et al. (1996)	L39082
BghPTR2	*B.graminis*	Aida droce et al.(2015)	AJ938049
CaCDR1	*C. albicans*	Prasad et al. (1995)	X77589
CaMDL1	*C. albicans*	McCreath (1997)	Y12327
CaOPT1	*C. albicans*	Lubkowitz et al. (1997)	U60973
CaPTR2	*C. albicans*	Basrai et al. (1995)	U09781
CtSDP1	*C.thermophilum*	This research	MH745073
CtSDP2	*C.thermophilum*	This research	MH745074
CtSDP3	*C.thermophilum*	This research	MH745075
dtpT	*L. lactis*	Hagting et al. (1994)	U05215
FgPTR2A	*F.graminearum*	Aida droce et al.(2017)	GN10354
FgPTR2B	*F.graminearum*	Aida droce et al.(2017)	GN103542
FgPTR2C	*F.graminearum*	Aida droce et al.(2017)	GN103538
FgPTR2D	*F.graminearum*	Aida droce et al.(2017)	ESU15443
MusMDRa	*M. musculus*	Gros et al. (1988)	J03398
OcPEPT1	*O. cuniculus*	Fei et al. (1994)	U06467
PcOPT5	*P.chrysosporium*	Quanju Xiang et al.(2013)	JX944672
RatPEPT1	*R. norvegicus*	Miyamoto et al. (1996)	D50664
ScMDL1	*S. cerevisiae*	Dean et al. (1994)	L16958
ScMDL2	*S. cerevisiae*	Dean et al. (1994)	L16959
ScPTR2	*S. cerevisiae*	Perry et al. (1994)	L11994
ScSTE6	*S. cerevisiae*	Kuchler et al. (1989)	M26376
ScYJL212C	*S. cerevisiae*	Walsh and Barrell (1996)	Z49487
ScYPR194C	*S. cerevisiae*	Walsh and Barrell (1996)	U25841
SpISP4	*S. pombe*	Sato et al. (1994)	P40900
TlSDP	*T.lanuginosus*	This research	MH745072
CgOPT1	*C.gloeosporioides*	Véronique Chagué et al.(2009)	FJ008981
NcIsp4	*N.crassa*		EAA35341.1
AoOPT	*A.oryzae*		BAE60512.1

Table S4. Average lifespan of randomly selected strains of *Chaetomium thermophilum* in three regions of Northeast of China

Species	Strain	Average lifespan ±standard deviation (days)	Sample source (isolated from)	Isolation region (Sample from)
Chaetomium thermophilum	Ct4	55.3±2.4	Cow dung	Changchun
Chaetomium thermophilum	Ct12	51.6±1.6	Decayed corn straw	Changchun
Chaetomium thermophilum	Ct33	48.4±5.3	Horse dung	Changchun
Chaetomium thermophilum	Ct24	37.0±0.9	Cow dung + rice straw	Changchun
Chaetomium thermophilum	Ct58	38.1±1.5	Rice straw compost	Changchun
Chaetomium thermophilum	Ct46	46.7±2.2	Chicken manure	Changchun
Chaetomium thermophilum	Ct1	45.1±1.0	Cow dung	Harbin
Chaetomium thermophilum	Ct42	39.5±2.6	Decayed corn straw	Harbin

Chaetomium thermophilum	Ct93	55.0±1.9	Horse dung	Harbin
Chaetomium thermophilum	Ct114	33.5±2.1	Cow dung + rice straw	Harbin
Chaetomium thermophilum	Ct56	58.3±4.7	Rice straw compost	Harbin
Chaetomium thermophilum	Ct146	54.8±2.0	Chicken manure	Harbin
Chaetomium thermophilum	Ct27	51.2±0.8	Cow dung	Dalian
Chaetomium thermophilum	Ct503	58.0±0.7	Decayed corn straw	Dalian
Chaetomium thermophilum	Ct442	39.6±4.1	Horse dung	Dalian
Chaetomium thermophilum	Ct15	47.3±2.3	Cow dung + rice straw	Dalian
Chaetomium thermophilum	Ct56	57.4±2.2	Rice straw compost	Dalian
Chaetomium thermophilum	Ct76	39.6±1.5	Chicken manure	Dalian

Note: Samples collected from the year 2006 to 2018, from which strains were isolated in our lab.

Figure S1

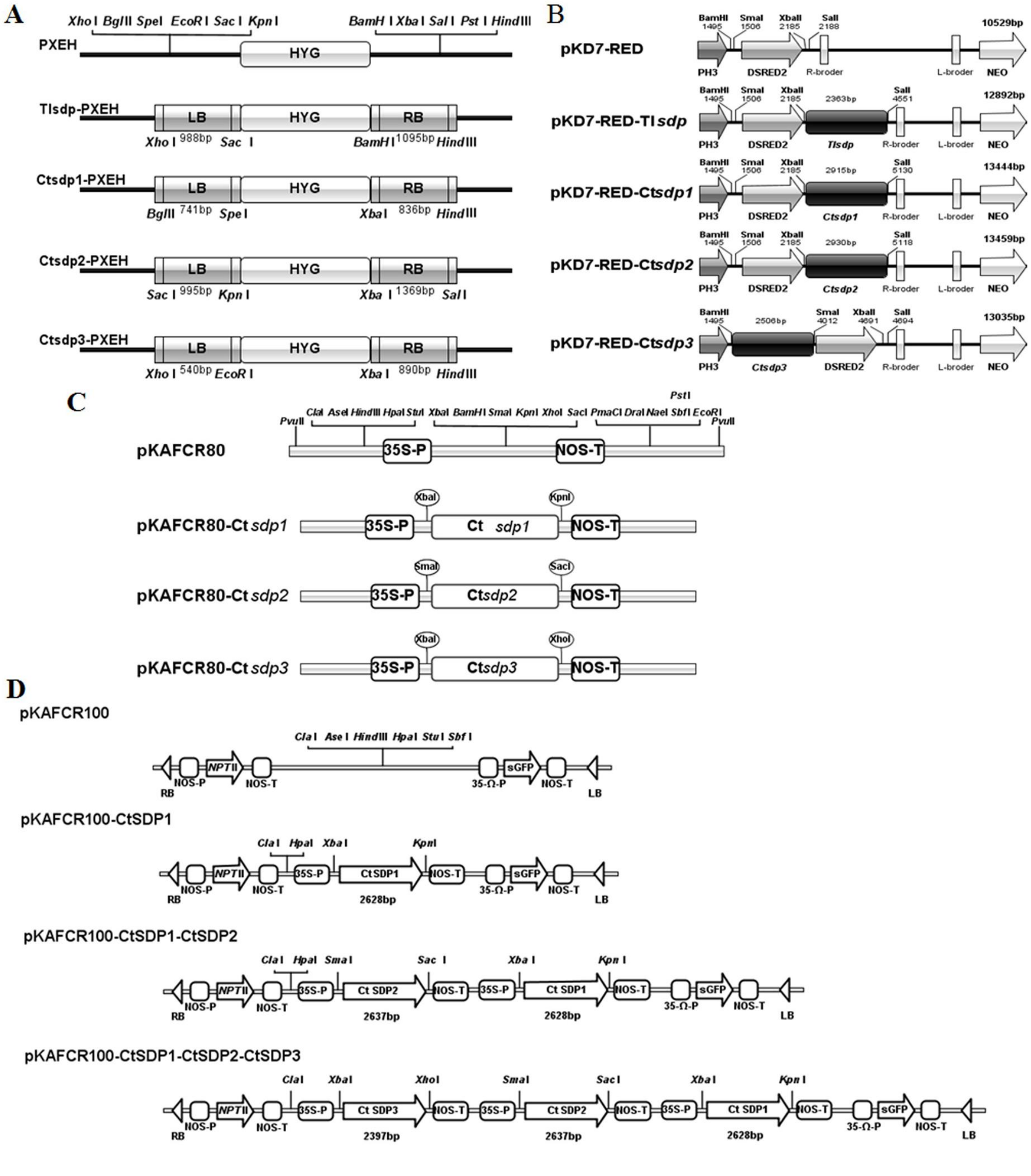
A
Xho I Bgl II Spe I EcoR I Sac I Kpn I
BamH I Xba I Sal I Pst I Hind III
PXEH
HYG
Tlsdp-PXEH
LB
HYG
RB
Xho I 988bp Sac I
BamH I 1095bp Hind III
Ctsdp1-PXEH
Bgl II 741bp Spe I
Xba I 836bp Hind III
Ctsdp2-PXEH
Sac I 995bp Kpn I
Xba I 1369bp Sal I
Ctsdp3-PXEH
Xho I 540bp EcoR I
Xba I 890bp Hind III
B
pKD7-RED
pKD7-RED-Tl sdp
pKD7-RED-Ct sdp1
pKD7-RED-Ct sdp2
pKD7-RED-Ct sdp3
PH3
DSRED2
R-broder
L-broder
NEO
10529bp
12892bp
13444bp
13459bp
13035bp
C
pKAFCR80
pKAFCR80-Ct sdp1
pKAFCR80-Ct sdp2
pKAFCR80-Ct sdp3
35S-P
NOS-T
D
pKAFCR100
pKAFCR100-CtSDP1
pKAFCR100-CtSDP1-CtSDP2
pKAFCR100-CtSDP1-CtSDP2-CtSDP3
2628bp
2637bp
2397bp

Figure S2

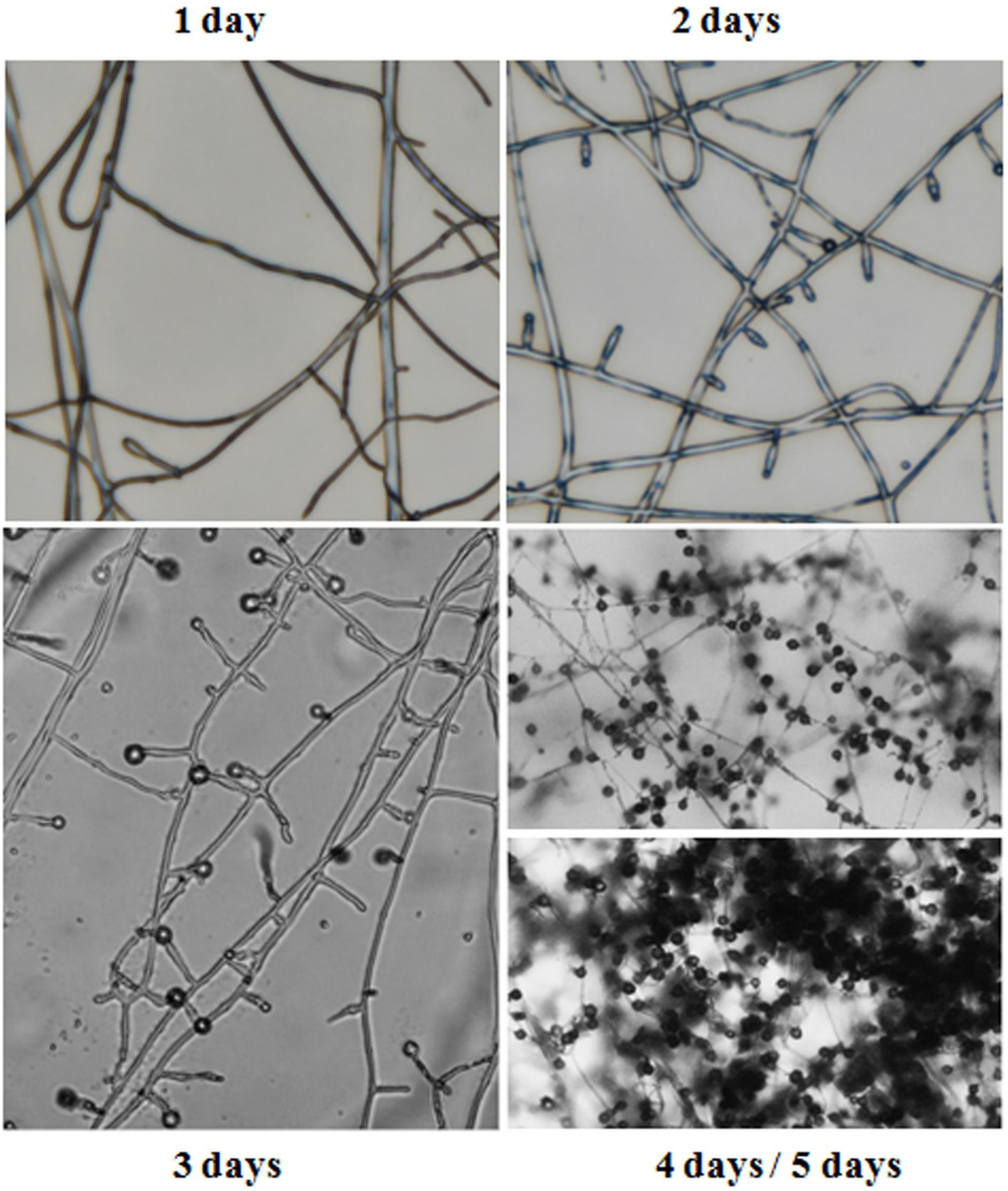

Figure S3

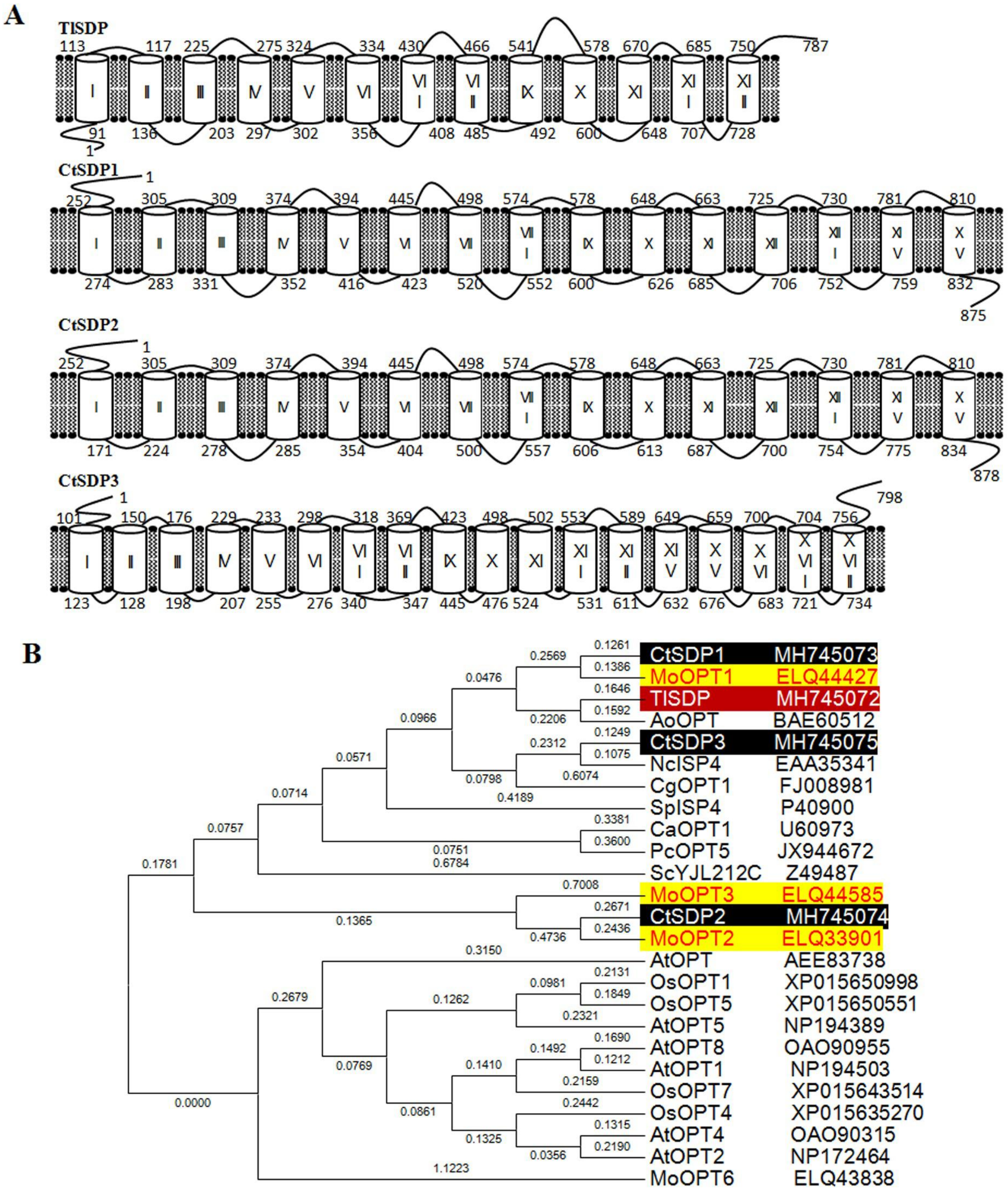
A
TlSDP
CtSDP1
CtSDP2
CtSDP3
B
CtSDP1 MH745073
MoOPT1 ELQ44427
TlSDP MH745072
AoOPT BAE60512
CtSDP3 MH745075
NcISP4 EAA35341
CgOPT1 FJ008981
SpISP4 P40900
CaOPT1 U60973
PcOPT5 JX944672
ScYJL212C Z49487
MoOPT3 ELQ44585
CtSDP2 MH745074
MoOPT2 ELQ33901
AtOPT AEE83738
OsOPT1 XP015650998
OsOPT5 XP015650551
AtOPT5 NP194389
AtOPT8 OAO90955
AtOPT1 NP194503
OsOPT7 XP015643514
OsOPT4 XP015635270
AtOPT4 OAO90315
AtOPT2 NP172464
MoOPT6 ELQ43838

FigureS4

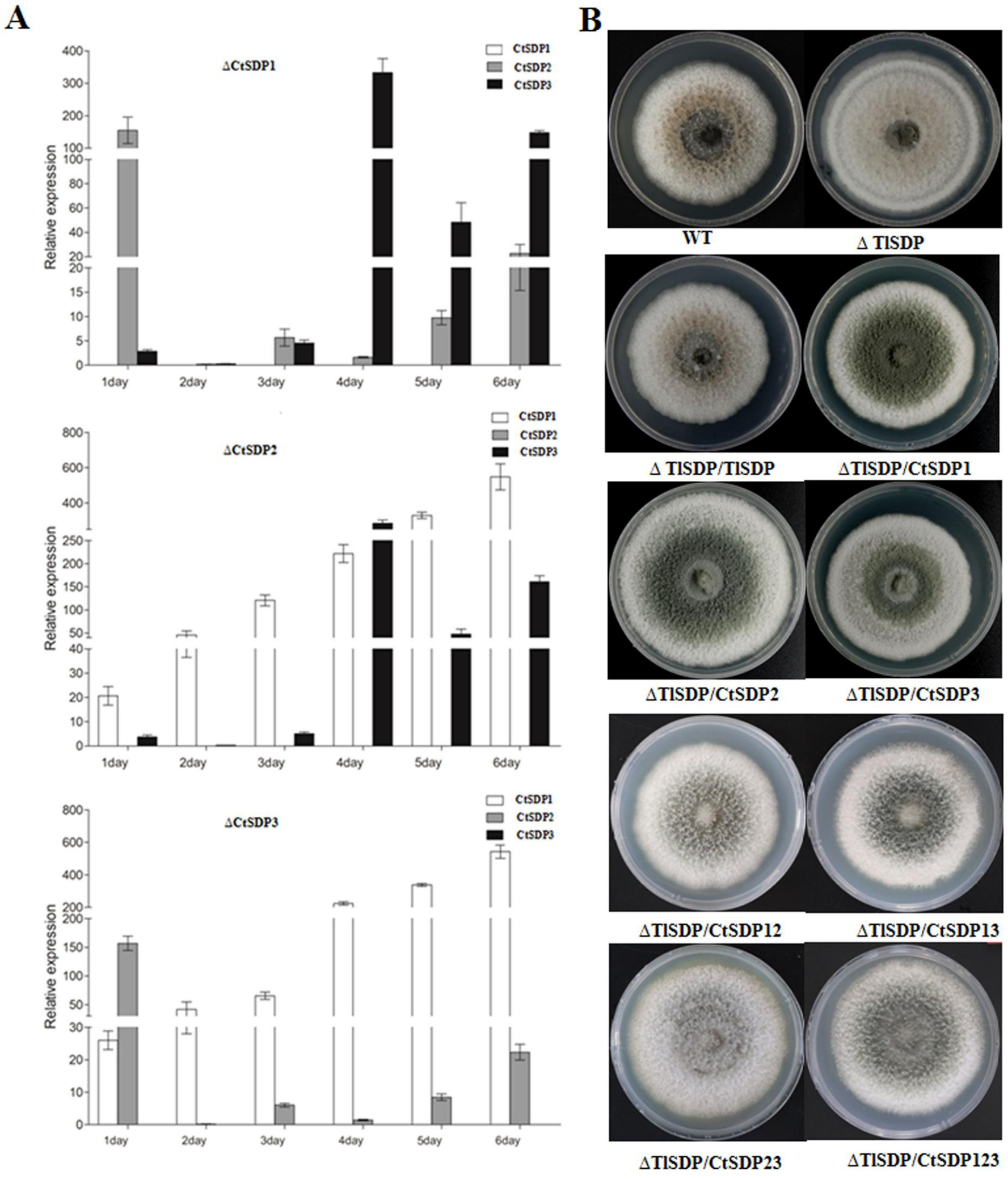
A
ΔCtSDP1
CtSDP1
CtSDP2
CtSDP3
Relative expression
1day
2day
3day
4day
5day
6day
ΔCtSDP2
CtSDP1
CtSDP2
CtSDP3
Relative expression
1day
2day
3day
4day
5day
6day
ΔCtSDP3
CtSDP1
CtSDP2
CtSDP3
Relative expression
1day
2day
3day
4day
5day
6day
B
WT
Δ TlSDP
Δ TlSDP/TlSDP
ΔTlSDP/CtSDP1
ΔTlSDP/CtSDP2
ΔTlSDP/CtSDP3
ΔTlSDP/CtSDP12
ΔTlSDP/CtSDP13
ΔTlSDP/CtSDP23
ΔTlSDP/CtSDP123

Figure S5

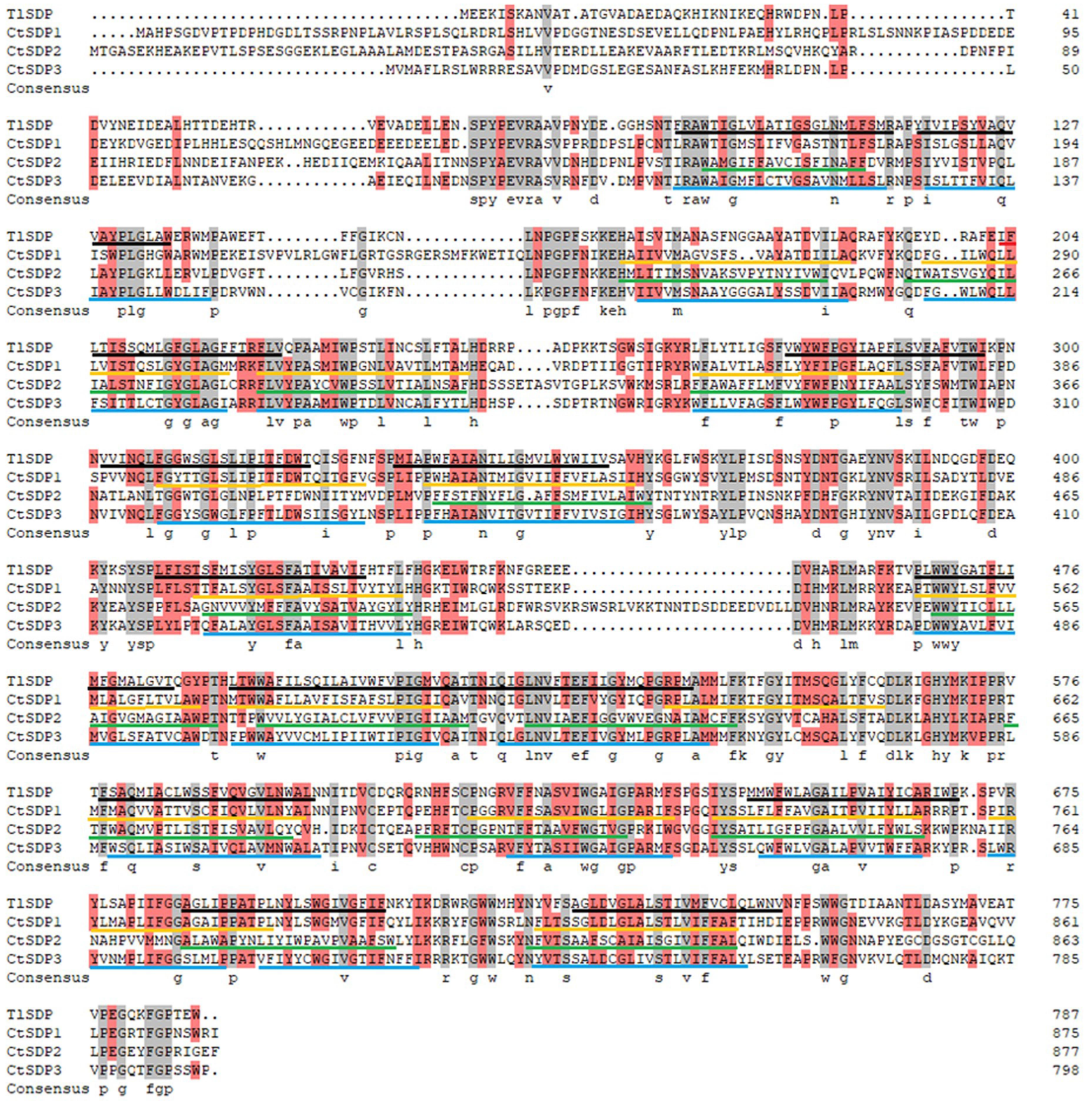

Figure S6

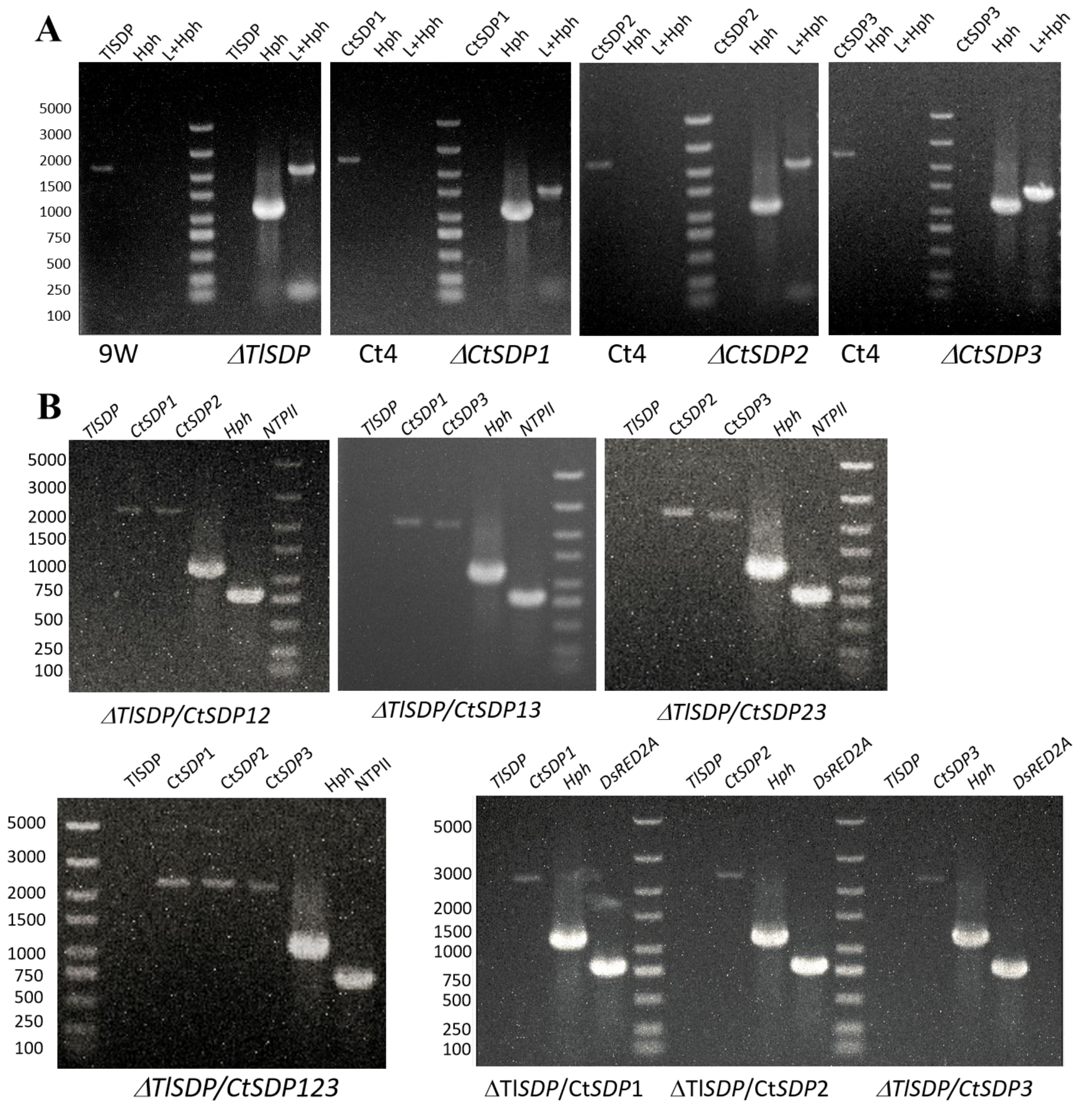

Figure S7

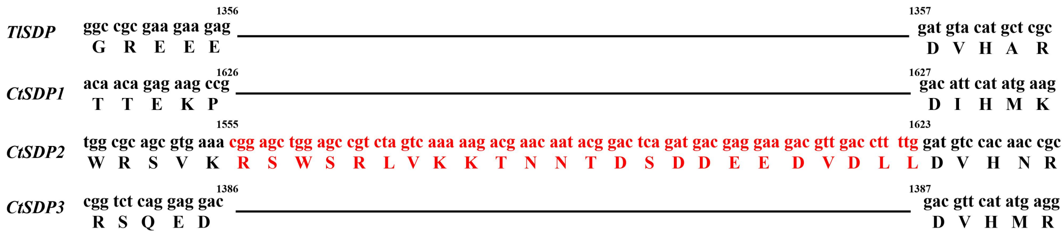

Supplement: Supplementary file 1 [file Data_Sheet_1.pdf]
